# A pedagogical walkthrough of computational modeling and simulation of Wnt signaling pathway using static causal models in MATLAB

**DOI:** 10.1186/s13637-016-0044-y

**Published:** 2016-08-08

**Authors:** Shriprakash Sinha

**Affiliations:** 104-Madhurisha Heights Phase 1, Risali, Bhilai, 490006 India

**Keywords:** Wnt signaling pathway, Bayesian network, Prior biological knowledge, Epigenetic information, Heterogeneous data integration, Hypothesis testing, Inference

## Abstract

Simulation study in systems biology involving computational experiments dealing with Wnt signaling pathways abound in literature but often lack a pedagogical perspective that might ease the understanding of beginner students and researchers in transition, who intend to work on the modeling of the pathway. This paucity might happen due to restrictive business policies which enforce an unwanted embargo on the sharing of important scientific knowledge. A tutorial introduction to computational modeling of Wnt signaling pathway in a human colorectal cancer dataset using static Bayesian network models is provided. The walkthrough might aid biologists/informaticians in understanding the design of computational experiments that is interleaved with exposition of the Matlab code and causal models from Bayesian network toolbox. The manuscript elucidates the coding contents of the advance article by Sinha (Integr. Biol. 6:1034–1048, 2014) and takes the reader in a step-by-step process of how (a) the collection and the transformation of the available biological information from literature is done, (b) the integration of the heterogeneous data and prior biological knowledge in the network is achieved, (c) the simulation study is designed, (d) the hypothesis regarding a biological phenomena is transformed into computational framework, and (e) results and inferences drawn using *d*-connectivity/separability are reported. The manuscript finally ends with a programming assignment to help the readers get hands-on experience of a perturbation project. Description of Matlab files is made available under GNU GPL v3 license at the Google code project on https://code.google.com/p/static-bn-for-wnt-signaling-pathway and https: //sites.google.com/site/shriprakashsinha/shriprakashsinha/projects/static-bn-for-wnt-signaling-pathway. Latest updates can be found in the latter website.

## Introduction

A tutorial introduction to computational modeling of Wnt signaling pathway in a human colorectal cancer dataset using static Bayesian network models is provided. This work endeavors to expound in detail the simulation study in MATLAB along with the code while explaining the concepts related to Bayesian networks. This is done in order to ease the understanding of beginner students and researchers in transition to computational signaling biology, who intend to work in the field of modeling of the signaling pathways. The manuscript elucidates (a) embedding of prior biological knowledge, (b) integration of heterogeneous information, (c) transformation of biological hypothesis into computational framework, and (d) design of the experiments, in a simple manner. This is interleaved with aspects of Bayesian network toolbox and MATLAB code so as to help readers get a feel of a project related to modeling of the pathway. Programming along with the exposition in the manuscript could clear up issues faced during the execution of the project.

**Fig. 1:**
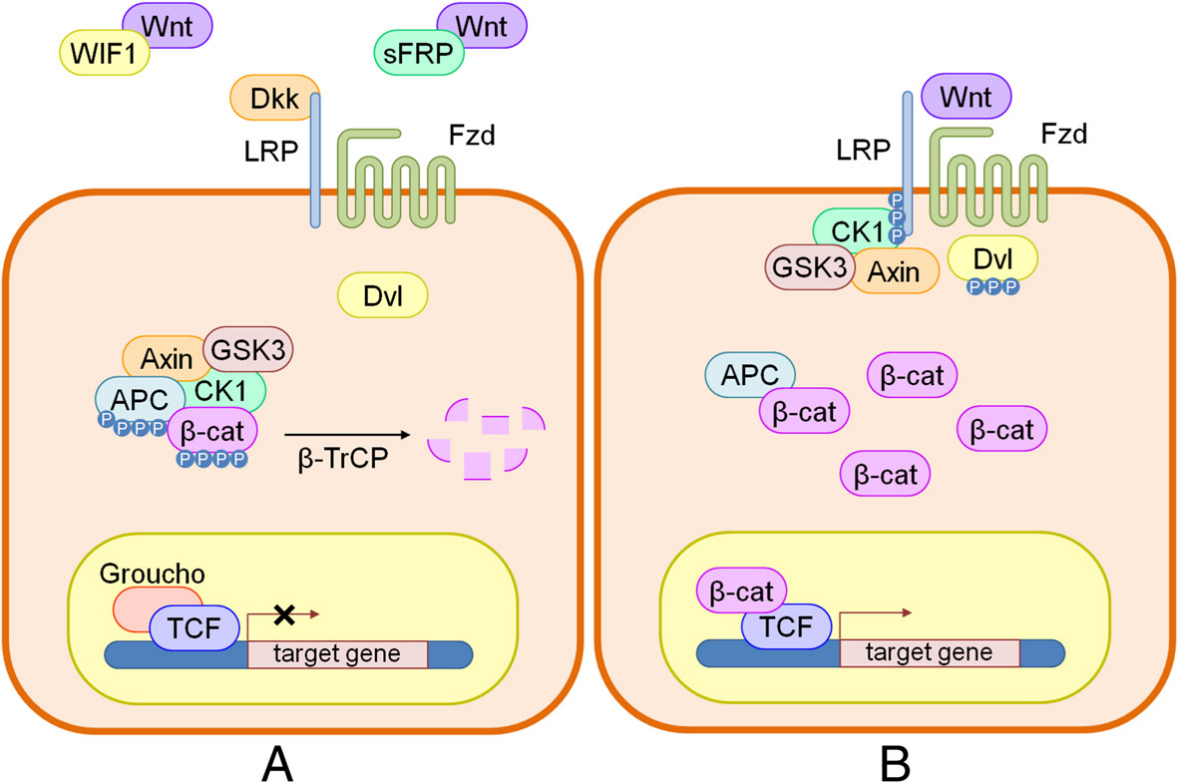
A cartoon of Wnt signaling pathway contributed by [3]. Part **a** represents the destruction of *β*-*catenin* leading to the inactivation of the Wnt target gene. Part **b** represents activation of Wnt target gene

This manuscript uses the contents of the advance article [1] as a basis to explain the workflow of a computational simulation project involving Wnt signaling pathway in human colorectal cancer (See Table 2 and Fig. 1 for description). The aim of [1] was to computationally test whether the activation of *β*-*catenin* and *T* *C* *F*4-based transcription complex always corresponds to the tumorous state of the test sample or not. To achieve this, the gene expression data provided by [2] was used in the computational experiments. Furthermore, to refine the model, prior biological knowledge related to the intra/extracellular factors of the pathway (available in literature) was integrated along with epigenetic information.

Section 4 of [1] has been reproduced for completeness in Tables 1, 2, 3, 4, 5, 6, and 7 in order. These tables provide introductory theory that will help in understanding the various aspects of the MATLAB code for modeling and simulation experiments that are explained later. More specifically, Table 1 gives an introduction to Bayesian networks. Tables 2 and 3 give a brief introduction to the canonical Wnt signaling pathway and the involved epigenetic factors, respectively. Table 4 gives a description of the three Bayesian network models developed with(out) prior biological knowledge. Tables 5 and 6 develop the network models with epigenetic information along with biological knowledge (Tables 8 and 9). Finally, Table 7 discusses a network model that has negligible prior biological knowledge. Code will be presented in typewriter font and functions in the text will be presented in sans serif. Reasons for taking certain approach and important information within the project are presented in small capitals.

**Table 1:** Bayesian networks from [1]

Bayesian networks. In reverse engineering methods for control networks [10] there exist many methods that help in the construction of the networks from the datasets as well as give the ability to infer causal relations between components of the system. A widely known architecture among these methods is the Bayesian network (BN). These networks can be used for causal reasoning or diagnostic reasoning or both. It has been shown through reasoning and examples in [11] that the probabilistic inference mechanism applied via Bayesian networks are analogous to the structural equation modeling in path analysis problems. Initial works on BNs in [12, 13] suggest that the networks only need a relatively small amount of marginal probabilities for nodes that have no incoming arcs and a set of conditional probabilities for each node having one or more incoming arcs. The nodes form the driving components of a network and the arcs define the interactive influences that drive a particular process. Under these assumptions of influences the joint probability distribution of the whole network or a part of it can be obtained via a special factorization that uses the concept of direct influence and through dependence rules that define d-connectivity/separability as mentioned in [14] and [15]. This is illustrated through a simple example in [11].
The Bayesian networks work by estimating the posterior probability of the model given the dataset. This estimation is usually referred to as the Bayesian score of the model conditioned on the dataset. Mathematically, let $\mathcal {S}$ represent the model given the data $\mathcal {D}$ and *ξ* is the background knowledge. Then according to the Bayes Theorem [16]:

Thus the Bayesian score is computed by evaluating the *posterior* distribution $\mathcal {P}(\mathcal {S}|\mathcal {D},\xi)$ which is proportional to the *prior* distribution of the model $\mathcal {P}(\mathcal {S}|\xi)$ and the *likelihood* of the data given the model $\mathcal {P}(\mathcal {D}|\mathcal {S},\xi)$. It must be noted that the background knowledge is assumed to be independent of the data. Next, since the evaluation of probabilities require multiplications a simpler way is to take logarithmic scores which boils down to addition. Thus, the estimation takes the form

Finally, the likelihood of the function can be evaluated by averaging over all possible local conditional distributions parameterized by *θ*_*i*_s that depict the conditioning of parents. This is equated via

Work on biological systems that make use of Bayesian networks can also be found in [17–21]. Bayesian networks are good in generating network structures and testing a targeted hypothesis which confine the experimenter to derive causal inferences [22]. But a major disadvantage of the Bayesian networks is that they rely heavily on the conditional probability distributions which require good sampling of datasets and are computationally intensive. On the other hand, these networks are quite robust to the existence of the unobserved variables and accommodate noisy datasets. They also have the ability to combine heterogeneous datasets that incorporate different modalities. In this work, simple static Bayesian network models have been developed with an aim to show how (a) incorporation of heterogeneous data can be done to increase prediction accuracy of test samples, (b) prior biological knowledge can be embedded to model biological phenomena behind the Wnt pathway in colorectal cancer, (c) to test the hypothesis regarding direct correspondence of active state of *β*-*catenin*-based transcription complex and the state of the test sample via segregation of nodes in the directed acyclic graphs of the proposed models, and (d) inferences can be made regarding the hidden biological relationships between a particular gene and the *β*-*catenin* transcription complex. This work uses Matlab-implemented BN toolbox from [4].

**Table 2:** Canonical Wnt pathway from [1]

Canonical Wnt signaling pathway. The canonical Wnt signaling pathway is a transduction mechanism that contributes to embryo development and controls homeostatic self-renewal in several tissues [8]. Somatic mutations in the pathway are known to be associated with cancer in different parts of the human body. Prominent among them is the colorectal cancer case [23]. In a succinct overview, the Wnt signaling pathway works when the Wnt ligand gets attached to the frizzled(*fzd*)/*LRP* coreceptor complex. *Fzd* may interact with the disheveled (*Dvl*) causing phosphorylation. It is also thought that Wnts cause phosphorylation of the *LRP* via casein kinase 1 (*C* *K*1) and kinase *G* *S* *K*3. These developments further lead to attraction of axin which causes inhibition of the formation of the degradation complex. The degradation complex constitutes of *axin*, the *β*-*catenin* transportation complex *APC*, *C* *K*1, and *G* *S* *K*3. When the pathway is active, the dissolution of the degradation complex leads to stabilization in the concentration of *β*-*catenin* in the cytoplasm. As *β*-*catenin* enters into the nucleus, it displaces the *Groucho* and binds with transcription cell factor *TCF*, thus instigating transcription of Wnt target genes. *Groucho* acts as lock on *TCF* and prevents the transcription of target genes which may induce cancer. In cases when the Wnt ligands are not captured by the coreceptor at the cell membrane, *axin* helps in the formation of the degradation complex. The degradation complex phosphorylates *β*-*catenin* which is then recognized by *F* *b* *o* *x*/*WD* repeat protein *β*−*T* *r* *C* *P*. *β*−*T* *r* *C* *P* is a component of ubiquitin ligase complex that helps in ubiquitination of *β*-*catenin*, thus marking it for degradation via the proteasome. Cartoons depicting the phenomena of Wnt activation are shown in Fig. 1 a, b, respectively.

**Table 3:** Epigenetic factors from [1]

Epigenetic factors. One of the widely studied epigenetic factors is methylation [24–26]. Its occurrence leads to decrease in the gene expression which affects the working of Wnt signaling pathways. Such characteristic trends of gene silencing like that of secreted frizzled-related proteins (*SFRP*) family in nearly all human colorectal tumor samples have been found at extracellular level [27]. Similarly, methylation of genes in the Dickkopf (*DKKx* [28, 29]), Dapper antagonist of catenin (*DACTx* [2]), and Wnt inhibitory factor-1 (*W* *I* *F*1 [30]) family are known to have a significant effect on the Wnt pathway. Also, histone modifications (a class of proteins that help in the formation of chromatin which packs the DNA in a special form [31]) can affect gene expression [32]. In the context of the Wnt signaling pathway, it has been found that *DACT* gene family shows a peculiar behavior in colorectal cancer [2]. *D* *A* *C* *T*1 and *D* *A* *C* *T*2 showed repression in tumor samples due to increased methylation while *D* *A* *C* *T*3 did not show obvious changes to the interventions. It is indicated that *D* *A* *C* *T*3 promoter is simultaneously modified by both the repressive and activating (bivalent) histone modifications ([2]).

**Table 4:** Bayesian Wnt pathway from [1]

**Fig. 2:**
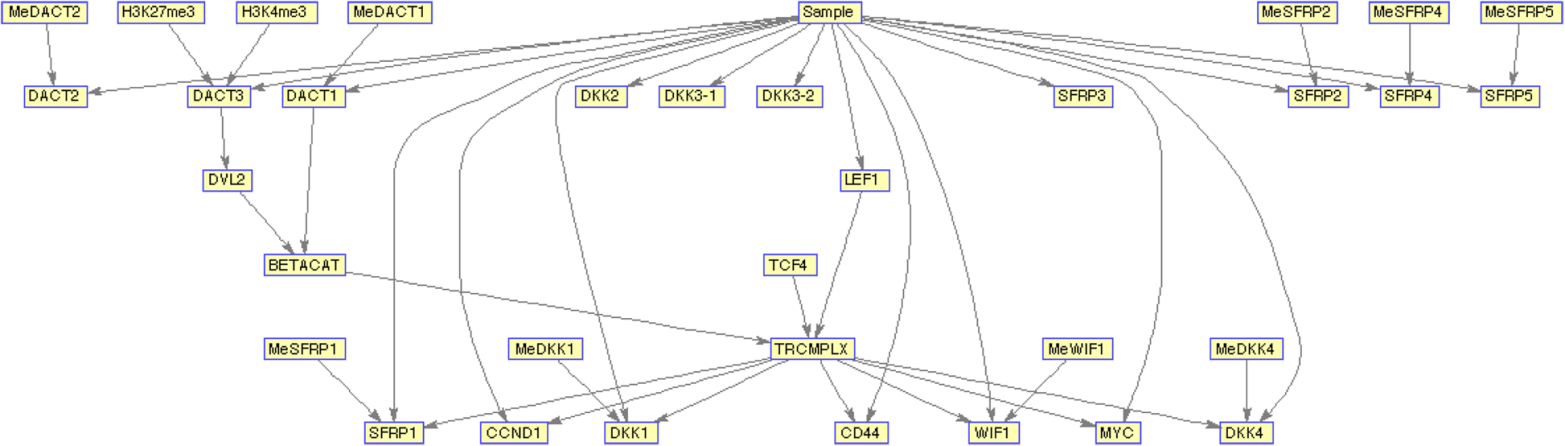
Influence diagram of $\mathcal {M}_{\text {PBK+EI}}$ contains partial prior biological knowledge and epigenetic information in the form of methylation and histone modification. In this model, the state of *Sample* is distinguished from state of *TRCMPLX* that constitutes the Wnt pathway

Bayesian Wnt pathway. Three static models have been developed based on particular gene set measured for human colorectal cancer cases [2]. Available epigenetic data for individual gene is also recorded. For sake of simplicity, the models are connoted as $\mathcal {M}_{\text {PBK+EI}}$ (model with prior biological knowledge (PBK) and epigenetic information (EI)), $\mathcal {M}_{\text {PBK}}$ (model with PBK only), and $\mathcal {M}_{\text {NB+MPBK}}$ (model with naive Bayes (NB) formulation and minimal PBK). All models are simple directed acyclic graphs (DAG) with nodes and edges. Figure 2 shows a detailed influence diagram of $\mathcal {M}_{\text {PBK+EI}}$ between the nodes and the edges. The nodes specify status of gene expression (*D* *K* *K*1, *D* *K* *K*2, *D* *K* *K*3-1, *D* *K* *K*3-2, *D* *K* *K*4, *D* *A* *C* *T*1, *D* *A* *C* *T*2, *D* *A* *C* *T*3, *S* *F* *R* *P*1, *S* *F* *R* *P*2, *S* *F* *R* *P*3, *S* *F* *R* *P*4, *S* *F* *R* *P*5, *W* *I* *F*1, *MYC*, *C* *D*44, *C* *C* *N* *D*1, and *L* *E* *F*1), methylation (*M* *e* *D* *A* *C* *T*1, *M* *e* *D* *A* *C* *T*2, *M* *e* *S* *F* *R* *P*1, *M* *e* *S* *F* *R* *P*2, *M* *e* *S* *F* *R* *P*4, *M* *e* *S* *F* *R* *P*5, *M* *e* *D* *K* *K*1, *M* *e* *D* *K* *K*4, and *M* *e* *W* *I* *F*1), histone marks for DACT3 (*H*3*K*27*m* *e*3 and *H*3*K*4*m* *e*3), transcription complex *TRCMPLX*, samples *Sample* and factors involved in formation of *TRCMPLX* like *β*-*catenin*, *T* *C* *F*4, and *L* *E* *F*1. Note that there were two recordings of gene expression *D* *K* *K*3 and thus were distinguished by *D* *K* *K*3−1 and *D* *K* *K*3−2. Some causal relations are based on prior biological knowledge and others are based on assumptions, elucidation of which follows in the next section.

**Table 5:** Network with PBK+EI from [1]

Network with PBK and EI the NB model [3] assumes that the activation (inactivation) of *β*-*catenin*-based transcription complex is equivalent to the fact that the sample is cancerous (normal). This assumption needs to be tested and in this research work, the two newly improvised models based on prior biological knowledge regarding the signaling pathway assume that sample prediction may not always mean that the *β*-*catenin*-based transcription complex is activated. These assumptions are incorporated by inserting another node of *Sample* for which gene expression measurements were available. This is separate from the *TRCMPLX* node that influences a particular set of known genes in the human colorectal cancer. For those genes whose relation with the *TRCMPLX* is currently not known or biologically affirmed, indirect paths through the *Sample* node to the *TRCMPLX* exist, technical aspect of which will be described shortly. Since all gene expressions have been measured from a sample of subjects, the expression of genes is conditional on the state of the *Sample*. Here, both tumorous and normal cases are present in equal amounts. The transcription factor *TRCMPLX* under investigation is known to operate with the help of interaction between *β*-*catenin* with *T* *C* *F*4 and *L* *E* *F*1 [9, 33]. It is also known that the regions in the TSS of *MYC* [34], *C* *C* *N* *D*1 [35], *C* *D*44 [36], *S* *F* *R* *P*1 [37], *W* *I* *F*1 [38], *D* *K* *K*1 [39], and *D* *K* *K*4 [40, 41] contain factors that have affinity to *β*-*catenin*-based *TRCMPLX*. Thus, expression of these genes are shown to be influenced by *TRCMPLX*, in Fig. 2.
Roles of *D* *K* *K*2 [42] and *D* *K* *K*3 [43, 44] have been observed in colorectal cancer but their transcriptional relation with *β*-*catenin*-based *TRCMPLX* is not known. Similarly, *S* *F* *R* *P*2 is known to be a target of *P* *a* *x*2 transcription factor and yet it affects the *β*-*catenin* Wnt signaling pathway [45]. Similarly, *S* *F* *R* *P*4 [46, 47] and *S* *F* *R* *P*5 [27] are known to have an effect on the Wnt pathway but their role with *TRCMPLX* is not well studied. *S* *F* *R* *P*3 is known to have a different structure and function with respect to the remaining *SFRPx* gene family [48]. Also, the role of *D* *A* *C* *T*2 is found to be conflicting in the Wnt pathway [49]. Thus, for all these genes whose expression mostly have an extracellular effect on the pathway and information regarding their influence on *β*-*catenin*-based *TRCMPLX* node is not available, an indirect connection has been made through the *Sample* node. This connection will be explained at the end of this section.

**Table 6:** Network with PBK+EI continued from [1]

**Fig. 3:**
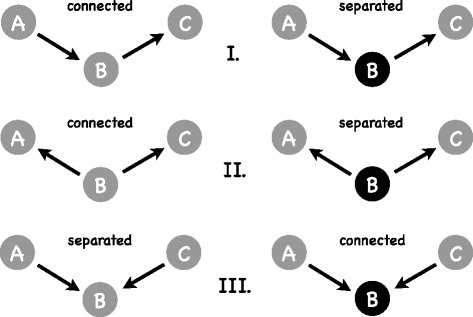
Cases for d-connectivity and d-separation. *Black* (*gray*) *circles* mean that evidence is available (not available) regarding a particular node

Network with PBK and EI continued … Lastly, it is known that concentration of *D* *V* *L*2 (a member of disheveled family) is inversely regulated by the expression of *D* *A* *C* *T*3 [2]. High *D* *V* *L*2 concentration and suppression of *D* *A* *C* *T*1 leads to increase in stabilization of *β*-*catenin* which is necessary for the Wnt pathway to be active [2]. But in a recent development [7], it has been found that expression of *D* *A* *C* *T*1 positively regulates *β*-*catenin*. Both scenarios need to be checked via inspection of the estimated probability values for *β*-*catenin* using the test data. Thus, there exists direct causal relations between parent nodes *D* *A* *C* *T*1 and *D* *V* *L*2 and child node, *β*-*catenin*. Influence of methylation (yellow hexagonal) nodes to their respective gene (green circular) nodes represent the effect of methylation on genes. Influence of histone modifications in *H*3*K*27*m* *e*3 and *H*3*K*4*m* *e*3 (blue octagonal) nodes to *D* *A* *C* *T*3 gene node represents the effect of histone modification on *D* *A* *C* *T*3. The *β*-*catenin* (blue square) node is influenced by concentration of *D* *V* *L*2 (depending on the expression state of *D* *A* *C* *T*3) and behavior of *D* *A* *C* *T*1. The aforementioned established prior causal biological knowledge is imposed in the BN model with the aim to computationally reveal unknown biological relationships. The influence diagram of this model is shown in Fig. 2 with nodes on methylation and histone modification. Another model $\mathcal {M}_{\text {PBK}}$ (not shown here) was developed excluding the epigenetic information (i.e., removal of nodes depicting methylation and histone modification as well as the influence arcs emerging from them) with the aim to check whether inclusion of epigenetic factors increases the cancer prediction accuracy.
In order to understand indirect connections further, it is imperative to know about *d-connectivity/separability*. In a BN model, this connection is established via the principle of *d-connectivity* which states that nodes are *connected* in a path when there exists no node in the path that has more than one incoming influence edge or there exists nodes in the path with more than one incoming influence edge which are observed (i.e., evidence regarding such nodes is available) [50]. Conversely, via principle of *d-separation*, nodes are *separated* in a path when there exists nodes in the path that have more than one incoming influence edge or there exists nodes in the path with at most one incoming influence edge which are observed (i.e., evidence regarding such nodes is available). Figure 3 represents three different cases of connectivity and separation between nodes $\mathcal {A}$ and $\mathcal {C}$ when the path between them passes through node $\mathcal {B}$. Connectivity or dependency exists between nodes $\mathcal {A}$ and $\mathcal {C}$ when (a) evidence is not present regarding node $\mathcal {B}$ in the left graphs of I and II in Fig. 3 or (b) evidence is present regarding node $\mathcal {B}$ in the right graph of III in Fig. 3.
Conversely, separation or independence exists between nodes $\mathcal {A}$ and $\mathcal {C}$ when (a) evidence is present regarding node $\mathcal {B}$ in the right graphs of I and II in Fig. 3 or (b) evidence is not present regarding node $\mathcal {B}$ in the left graph of III in Fig. 3. It would be interesting to know about the behavior of *TRCMPLX*, given the evidence of state of *S* *F* *R* *P*3. To reveal such information, paths must exist between these nodes. It can be seen that there are multiple paths between *TRCMPLX* and *S* *F* *R* *P*2 in the BN model in Fig. 2. These paths are enumerated as follows:
1. *S* *F* *R* *P*3, *Sample*, *S* *F* *R* *P*1, *TRCMPLX*
2. *S* *F* *R* *P*3, *Sample*, *D* *K* *K*1, *TRCMPLX*
3. *S* *F* *R* *P*3, *Sample*, *W* *I* *F*1, *TRCMPLX*
4. *S* *F* *R* *P*3, *Sample*, *C* *D*44, *TRCMPLX*
5. *S* *F* *R* *P*3, *Sample*, *D* *K* *K*4, *TRCMPLX*
6. *S* *F* *R* *P*3, *Sample*, *C* *C* *N* *D*1, *TRCMPLX*
7. *S* *F* *R* *P*3, *Sample*, *MYC*, *TRCMPLX*
8. *S* *F* *R* *P*3, *Sample*, *L* *E* *F*1, *TRCMPLX*
9. *S* *F* *R* *P*3, *Sample*, *D* *A* *C* *T*3, *D* *V* *L*2, *β*-*catenin*, *TRCMPLX*
10. *S* *F* *R* *P*3, *Sample*, *D* *A* *C* *T*1, *β*-*catenin*, *TRCMPLX*
Knowledge of evidence regarding nodes of *S* *F* *R* *P*1 (path 1), *D* *K* *K*1 (path 2), *W* *I* *F*1 (path 3), *C* *D*44 (path 4), *D* *K* *K*4 (path 5), *C* *C* *N* *D*1 (path 6), and *MYC* (path 7) makes *Sample* and *TRCMPLX* dependent or d-connected. Further, no evidence regarding state of *Sample* on these paths instigates dependency or connectivity between *S* *F* *R* *P*3 and *TRCMPLX*. On the contrary, evidence regarding *L* *E* *F*1, *D* *A* *C* *T*3, and *D* *A* *C* *T*1 makes *Sample* (and child nodes influenced by *Sample*) independent or d-separated from *TRCMPLX* through paths (8) to (10). Due to the dependency in paths (1) to (7) and the given state of *S* *F* *R* *P*3 (i.e., evidence regarding it being active or passive), the BN uses these paths during inference to find how *TRCMPLX* might behave in normal and tumorous test cases. Thus, exploiting the properties of d-connectivity/separability, imposing a biological structure via simple yet important prior causal knowledge and incorporating epigenetic information, BN helps in inferring many of the unknown relation of a certain gene expression and a transcription complex.

**Table 7:** Network with NB+MPBK from [1]

**Fig. 4:**
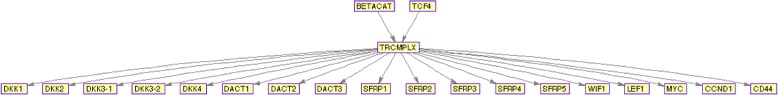
Influence diagram of $\mathcal {M}_{\text {NB+MPBK}}$ is a naive Bayes model that contains minimal prior biological knowledge. In this model, the state of *TRCMPLX* is assumed to be indicate whether the sample is cancerous or not

Network with minimal PBK. Lastly, a naive Bayes model $\mathcal {M}_{\text {NB+MPBK}}$ with minimal biological knowledge based on [3] model was also developed with an aim to check if the assumed hypothesis that activation state of *TRCMPLX* is the same as sample being cancerous is correct. In this model, all gene expressions are assumed to be transcribed via the *β*-*catenin*-based *TRCMPLX* and thus causal arcs exist from *TRCMPLX* to different gene nodes. The complex itself is influenced by *β*-*catenin* and *T* *C* *F*4 only. Such models can be used for prediction purpose but are not useful in revealing hidden biological relationships as no or minimal prior biological information is imposed on the naive Bayes model. Figure 4 shows the naive Bayes model.

**Table 8:** Conditional probability tables for nodes (excluding gene expression) of $\mathcal {M}_{\text {PBK+EI}}$

		Conditional probability table for nodes	
Node	Parents	Cpt values rep.	Node states
*Sample*	-	[0.50 0.50] ^*T*^	[n t]
*T* *C* *F*4	-	[0.10 0.90] ^*T*^	[ia a]
*D* *V* *L*2	*D* *A* *C* *T*3	[0.01 0.99; 0.99 0.01] ^*T*^	[lc hc]
*β*-*catenin*	*D* *A* *C* *T*1,	[0.99 0.99 0.99 0.01;	[lc hc]
	*D* *V* *L*2	0.01 0.01 0.01 0.99] ^*T*^	
*TRCMPLX*	*T* *C* *F*4, *L* *E* *F*1,	[0.99*ones(1,7) 0.01;	[ia a]
	*β*-*catenin*	0.01*ones(1,7) 0.99] ^*T*^	
*M* *e* *D* *A* *C* *T*1	-	[0.8370 0.1630] ^*T*^	[nm m]
*M* *e* *D* *A* *C* *T*2	-	[0.3376 0.6624] ^*T*^	[nm m]
*M* *e* *W* *I* *F*1	-	[0.1667 0.8333] ^*T*^	[nm m]
*M* *e* *S* *F* *R* *P*1	-	[0.6316 0.3684] ^*T*^	[nm m]
*M* *e* *S* *F* *R* *P*2	-	[0.6316 0.3684] ^*T*^	[nm m]
*M* *e* *S* *F* *R* *P*4	-	[0.8572 0.1428] ^*T*^	[nm m]
*M* *e* *S* *F* *R* *P*5	-	[0.7500 0.2500] ^*T*^	[nm m]
*H*3*K*27*m* *e*3	-	[0.2391 0.7609] ^*T*^	[ia a]
*H*3*K*4*m* *e*3	-	[0.3661 0.6339] ^*T*^	[ia a]

Notations in the table mean the following “-” implies no parents exist for the particular node; “n” - normal, “t” - tumorous, “ia” - inactive, “a” - active, “lc” - low concentration, “hc” - high concentration, “nm” - non-methylated, “m” - methylation

**Table 9:** Conditional probability tables for gene nodes of $\mathcal {M}_{\text {PBK+EI}}$

		Conditional probability table for nodes
Node	Parents	Cpt values rep.
*L* *E* *F*1	*Sample*	[0.84 0.16; 0.16 0.84] ^*T*^
*MYC*	*Sample*,	[0.94 0.89 0.78 0.31;
	*TRCMPLX*	0.06 0.11 0.22 0.69] ^*T*^
*C* *C* *N* *D*1	*Sample*,	[0.95 0.89 0.81 0.28;
	*TRCMPLX*	0.06 0.11 0.18 0.72] ^*T*^
*C* *D*44	*Sample*,	[0.93 0.90 0.67 0.42;
	*TRCMPLX*	0.07 0.10 0.33 0.58] ^*T*^
*D* *K* *K*1	*Sample*,	[0.95 0.93 0.07 0.05 0.77 0.60 0.40 0.23;
	*M* *e* *D* *K* *K*1,	0.05 0.07 0.93 0.95 0.23 0.40 0.60 0.76] ^*T*^
	*TRCMPLX*	
*D* *K* *K*2	*Sample*	[0.40 0.60; 0.60 0.40] ^*T*^
*D* *K* *K*3-1	*Sample*	[0.36 0.64; 0.64 0.36] ^*T*^
*D* *K* *K*3-2	*Sample*	[0.56 0.44; 0.44 0.56] ^*T*^
*D* *K* *K*4	*Sample*,	[0.94 0.88 0.82 0.28;
	*TRCMPLX*	0.06 0.11 0.18 0.72] ^*T*^
*D* *A* *C* *T*1	*Sample*,	[0.56 0.74 0.26 0.44;
	*M* *e* *D* *A* *C* *T*1	0.44 0.26 0.74 0.56] ^*T*^
*D* *A* *C* *T*2	*Sample*,	[0.60 0.71 0.29 0.40;
	*M* *e* *D* *A* *C* *T*2	0.40 0.29 0.71 0.60] ^*T*^
*D* *A* *C* *T*3	*Sample*,	[0.88 0.88 0.12 0.88 0.88 0.88 0.12 0.88;
	*H*3*K*27*m* *e*3,	0.12 0.12 0.88 0.12 0.12 0.12 0.88 0.12] ^*T*^
	*H*3*K*4*m* *e*3	
*S* *F* *R* *P*1	*Sample*,	[0.88 0.98 0.02 0.12 0.20 0.96 0.04 0.80;
	*M* *e* *S* *F* *R* *P*1,	0.12 0.02 0.98 0.88 0.80 0.04 0.96 0.20] ^*T*^
	*TRCMPLX*	
*S* *F* *R* *P*2	*Sample*,	[0.31 0.88 0.11 0.69;
	*M* *e* *S* *F* *R* *P*2	0.69 0.11 0.89 0.31] ^*T*^
*S* *F* *R* *P*3	*Sample*	[0.20 0.80; 0.80 0.20] ^*T*^
*S* *F* *R* *P*4	*Sample*,	[0.71 0.60 0.40 0.29;
	*M* *e* *S* *F* *R* *P*4	0.29 0.40 0.60 0.71] ^*T*^
*S* *F* *R* *P*5	*Sample*,	[0.31 0.89 0.11 0.69;
	*M* *e* *S* *F* *R* *P*5	0.69 0.11 0.89 0.31] ^*T*^
*W* *I* *F*1	*Sample*,	[0.96 0.91 0.09 0.04 0.85 0.47 0.56 0.15;
	*M* *e* *W* *I* *F*1,	0.04 0.09 0.91 0.96 0.15 0.53 0.47 0.85] ^*T*^
	*TRCMPLX*	

The state of the gene nodes remains [ia a], i.e., “ia” - inactive or “a” - active [ia a]. Note that these values are from one iteration of the 2-holdout experiment

## Motivation

### The project and issues involved

Drafting a manuscript that contains a pedagogical outlook of all the theory and the MATLAB code is a challenging task. This is because the background work of coding in a modeling and simulation project faces several issues that need to be overcome. Here, a few of these issues are discussed, but they are by no means complete. Some of the issues might be general across different computational biology projects while others might be more specific to the current project.

The advanced article of [1] contains three different network models, one of which is the naive Bayes model. The implemented naive Bayes model in [1] is a simplification of the primitive model proposed in [3]. The other two models are improvements over the naive Bayes model which incorporate prior biological knowledge. This manuscript describes the implementation of these models using a single colorectal cancer dataset. The reason for doing this was to test the effectiveness of incorporating prior biological knowledge gleaned from literature study of genes related to the dataset as well as test a biological hypothesis from a computational point of view. The main issues that one faces in this project are (a) finding biological causal relations from already published wet lab experiments, (b) designing the graphical network from biological knowledge, (c) translating the measurements into numerical values that form the prior beliefs of nodes in the network, (d) estimating the conditional probability values for nodes with parents, (e) framing the biological hypothesis into computational framework, (f) choosing the design of the learning experiment depending on the type of data, (g) inferring the hidden biological relations after the execution of the Bayesian network inference engine, and finally (h) presenting the results in a proper format via statistical significance tests.

### Biological causal relations

Often, biological causal relations are embedded in the literature pertaining to wet lab experiments in molecular biology. These relations manifest themselves as discovery/confirmation of one or multiple factors affecting the expression of a gene by either inhibiting or activating it. In context of the dataset used in the current work, the known causal relations were gleaned from review of such literature for each intra/extracellular factor involved in the pathway. The arcs in the Bayesian networks with prior biological knowledge encode these causal semantics. For those factors whose relations have not been confirmed but known to be involved in the pathway, the causal arcs were segregated via a latent variable that is introduced into the Bayesian network. The latent variable in the form of “sample” (see Fig. 2) is extremely valuable as it connects the factors whose relations have not been confirmed till now, to factors whose influences have been confirmed in the pathway. Detailed explanation of the connectivity can be found in Table 6. Also, the introduction of latent variable in a causal model opens an avenue to assume the presence of measurements that haven’t been recorded. Intuitively, for cancer samples the hidden measurements might be different from those for normal samples. The connectivity of factors through the variable provides an important route to infer biological relations. Finally, the problem with such models is that it is static in nature. This means that the models represent only a snapshot of the connectivity in time, which is still an important information for further research. By using time course data it might be possible to reveal greater biological information dynamically. The current work lacks in this endeavor and considers the introduction of time course-based dynamic models for future research work.

### Bayesian networks, parameter estimation, biological hypothesis

Bayesian networks are probabilistic graphical models that encode causal semantics among various factors using arcs and nodes. The entire network can represent a framework for a biological pathway and can be used to predict, explore or explain certain behaviors related to the pathway (See Tables 5 and 6 and Fig. 3 for description). As previously stated, the directionality of the arcs define the causal influence while the nodes represent the involved factors. Also, it is not just the arcs and nodes that play a crucial role. Information regarding the strength of the belief in a factor’s involvement is encoded as prior probability (priors) or conditional probability values. Estimation of these probabilities are either via expert’s knowledge or numerical estimations in the form of frequencies gleaned from measurements provided in the literature from wet lab experiments. In this project, the nodes are discrete in nature. Since the models are a snapshot in time, discrete nodes help in encoding specific behavior in time. Here, discretization means defining the states in which a factor can be (say a gene expression is on or off, or methylation is on or off, etc). As stated above, this leads to loss of continuous information revealed in time series data.

As depicted in the model in Fig. 2 and described in Tables 5 and 6, to test one of the biological hypothesis that *TRCMPLX* is not always switched on (off) when the sample is tumorous (normal), the segregation of *TRCMPLX* node from *Sample* node was made in [1]. Primitive models of the Naive Bayes network assume direct correspondence of *TRCMPLX* and *Sample* as depicted in [1] and [3]. The segregated design helps in framing the biological hypothesis into computational framework. The basic factor in framing the biological hypothesis to a computational framework requires knowledge of how the known factors of the pathway are involved, how the unknown factors need to be related to the known factors and finally intuitive analysis of the design of the model (for static data). Note that the model is a representation and not complete. Larger datasets will complicate the model and call for more efficient designs.

### Choice of data

In a data dependent model, the data guides the working of the model and the results obtained depend on the design of the experiments to be conducted on the data. The current work deals with gene expression data from 24 samples each of human colorectal tumor and matched normal mucosa. Different expression values across the samples are recorded for total of 18 genes known to work at different cellular regions in the pathway. This dataset from [2] was specifically chosen because it covers a small range of important genes whose expression measurements are influenced by epigenetic factors, crucial information about which is enough to build a working prototype model. Also, this dataset though not complete, contains enough information to design small computational experiments to test certain biological hypothesis which will be seen later.

From one point of view, this paper’s analysis is essentially an exercise in biomarker validation: do the genes selected for follow-up predict tumor status of tissue samples? In the implementation used here, they do not do so with full reliability. This raises the question of the validity of using the small subset of the WNT pathway chosen as a predictive biomarker of tumor status—This is true! That is why the idea was to segregate the node *Sample* from *TRCMPLX* and check the biological hypothesis whether the active (inactive) state of transcription complex is directly related to the sample being tumorous (normal), from a computational perspective. It was found that it is not necessary that *TRCMPLX* is switched on (off) when the sample is tumorous (normal) given a certain gene expression. By developing a biologically inspired model on this small dataset, one is able to detect if the predictions always point to the biological phenomena or not. In this case, the sample being tumorous or normal given the gene expression evidence is based on a Naive Bayes model (similar to [3]) which does not incorporate prior biological knowledge. It is not the small dataset always that matters but how the network is designed that matters. The status of a sample being tumorous/normal might be inferred in a better way if the prior biological knowledge regarding the pathway was also incorporated and the dominant factor like the activation of transcription complex along with established biomarkers was studied. Sinha [1] gave an improvement over the model implemented in [3] for this very reason.

### Design of experiments

A two holdout experiment is conducted in order to reduce the bias induced by unbalanced training data. From a machine learning perspective, this bias is removed by selecting one sample from normal and one sample from tumor for testing purpose and the remaining samples to form the training dataset. The procedure of selection is repeated for all possible combinations of a normal sample and a tumor sample. What happens is that the training data remains balanced and each pair of test sample (one normal and one tumor) gets evaluated for prediction of the label. Repetitions of a normal (tumor) sample across test pairs give equal chance for each of the tumor (normal) sample to be matched and tested.

### Inference and statistical tests

The inference of the biological relations is done by feeding the the evidence into the model and computing the conditional probability of the effect of a factor(s) given the evidence. Note that the Bayesian network used in the BNT toolbox by [4] uses the two-pass junction tree algorithm. In the first pass, the Bayesian network engine is created and initialized with prior and estimated probabilities for the nodes in the network. In the second pass, after feeding in the evidence for some of the nodes, the parameters for the network are recomputed. It is these recomputed parameters that give insight into the hidden biological relations based on the design of the network as well as the use of the principle of d-connectivity/separability. Since the computed conditional probabilities may change depending on the quality of evidence per test sample that is fed to the network, statistical estimates are deduced and receiver operator curves (ROC) along with respective their area under the curve (AUC) are plotted. These estimates give a glimpse of the quality of predictions. Apart from this, since a distribution of predictions is generated via 2-holdout experiment, Kolmogorov-Smirnov test is employed to check the statistical significance between the distributions. The significance test helps in comparing the prediction results for hypothesis testing in different models and thus point to the effectiveness of the models regarding biological interpretations.

This non-parametric test will reject the null hypothesis when distributions differ in shape. The author notes that his more complex biologically inspired models give significant KS test *p* values when comparing predictions of the *β*-*catenin* transcription factor complex state and the tumor/non-tumor status of the samples. While the result is interesting, the KS test adds little information on interpretation. Are the biological models incorrect? Are the predictions produced using faulty assumptions? Are false positives or false negatives more frequent, and if so why?

Biological models might be lacking in biological information and correctness depends on how the model is designed. This does not mean that the inferences are wrong and the assumptions are faulty. The differences in the distribution is due to the prior biological knowledge that has been incorporated into the models. So indirectly, the KS test points to the significance of adding the biological data. While using the naive Bayes model (from [3]), it was found that the prediction accuracy was almost 100 *%*. But w.r.t. issue raised regarding the biomarker prediction earlier, the accuracy value drops due to the model complexity and correct biological inferences can be made. From the Bayesian perspective, the numerical value represents a degree of belief in an event and the 100 *%* prediction accuracy might not capture the biological phenomena as well as the influence of the biomarker properly from the naive Bayes model with minimal prior biological knowledge in [3] and [1]. Thus, KS test gives an indirect indication regarding the significance of using the prior biological knowledge in comparison to the negligible knowledge while designing the models.

### MATLAB and Bayesian network toolbox

The choice of MATLAB was made purely because of its ability to handle various types of data structures which can be used for fast prototype building. Also, the BNT toolbox is freely available and provides most of the functions necessary to deal with the design of the Bayesian network models of different types (both static and dynamic). There are many packages freely available in *R* that could be used for development of these projects, but they lack the level of details that the BNT toolbox provides. The downside of the BNT toolbox is that one needs a MATLAB license. Finally, the BNT toolbox can be downloaded from https://code.google.com/p/bnt/. Instructions for installations as well as how to use the package is available in the website. The material from [1] has been made available in the Google drive https://drive.google.com/folderview?id=0B7Kkv8wlhPU-T05wTTNodWNydjA&usp=sharing. This contains the individual files, contents of which are used in this manuscript. The drive and its contents can be accessed via the URLs mentioned earlier in the abstract. To ease the understanding of the know-how-it-works of BNT toolbox, the drive contains two files namely *sprinkler_rain_script.m* and *sprinkler_rain.mat*. The former contains code from BNT toolbox in a procedural manner and the latter contains the saved results after running the script. As a toy example, these can be used for quick understanding.

An important point of observation—while executing the code—if the chunks of code are not easy to follow, then please use the MATLAB facility of debugging by setting up *breakpoints* and a range of functions starting with prefix DB. Note that the breakpoints appear as solid red dots on the left hand side of the MATLAB editor when being used. When the code is running, solid green arrows stop at these breakpoints and let the user analyze the query of interest. More help is available on Internet as well as via the MATLAB help command.

## Modeling and simulation

### Data collection and estimation

An important component of this project is the Bayesian network toolbox provided by [4] and made freely available for download on https://code.google.com/p/bnt/ as well as a MATLAB license. Instructions for installations are provided on the mentioned website. To begin the project, one can make a directory titled *temp* with a subdirectory named *data* and transfer the *geneExpression.mat* file into *data*.



This.mat file contains expression profiles from [2] for genes that play a role in Wnt signaling pathway at an intra/extracellular level and are known to have inhibitory effect on the Wnt pathway due to epigenetic factors. For each of the 24 normal mucosa and 24 human colorectal tumor cases, gene expression values were recorded for 14 genes belonging to the family of *SFRP*, *DKK*, *W* *I* *F*1, and *DACT*. Also, expression values of established Wnt pathway target genes like *L* *E* *F*1, *MYC*, *C* *D*44, and *C* *C* *N* *D*1 were recorded per sample.

The directory *temp* also contains some of the.m files, parts of the contents of which will be explained in the order of execution of the project. The main code begins with a script titled *twoHoldOutExp.m* (Note that the original unrefined file is under the name *twoHoldOutExp-original.m*). This script contains the function twoHoldOutExp which takes two arguments named eviDence and model. eviDence implies the evidence regarding “ge” for gene evidence, “me” for methylation, “ge+me” for both gene and methylation, while model implies the network model that will be used for simulation. Sinha [1] uses three different models, i.e., “t1” or $\mathcal {M}_{\text {PBK+EI}}$ that contains prior biological knowledge as well as epigenetic information, “t2” or $\mathcal {M}_{\text {PBK}}$ that contains only prior biological knowledge, and, finally, “p1” or $\mathcal {M}_{\text {NB+MPBK}}$ that is a modified version of the naive Bayes framework from [3]. On the MATLAB command prompt, one can type the following



The code begins with the extraction of data from the gene expression matrix by reading the *geneExpression.mat* file via the function readCustomFile in the *readCustomFile.m* and generates the following variables as the output: (1) uniqueGenes—name of genes gleaned from the file, (2) expressionMatrix—2D matrix containing the gene expression per sample data, (3) noGenes—total number of genes available, (4) noSamples—total number of samples available, (5) groundTruthLabels—original labels available from the files, and (6) transGroundTruthLabels—labels transformed into numerals. 



### Assumed and estimated probabilities from literature

Next, the probability values for some of the nodes in the network is loaded depending on the type of the network. Why these assumed and estimated probabilities have been addressed in the beginning of the computation experiment is as follows. It can be seen that the extra/intracellular factors affecting the Wnt pathway in the dataset provided by [2] contain some genes whose expression is influenced by epigenetic factors mentioned in Table 3. Hence, it is important to tabulate and store prior probability values for known epigenetic biological factors that influence the pathway. Other than the priors for epigenetic nodes, priors for some of the nodes that are a major component of the pathway but do not have data from prior approximation, are assumed based on expert knowledge. Once estimated or assumed based on biological knowledge, these probabilities need not be recomputed and are thus stored in proper format at the beginning of the computational experiment.

The estimation of prior probabilities is achieved through the function called dataStorage in the file *dataStorage.m*. The function takes the name of the model as an input argument and returns the name of the file called *probabilities.mat* in the variable filename. The.mat file contains all the assumed and computed probabilities of nodes for which data is available and is loaded into the *workspace* of the MATLAB for further use. The workspace is an area which stores all the current variables with their assigned instances such that the variables can be manipulated either interactively via command prompt or from different functions.



$\mathcal {M}_{\text {PBK+EI}}$ (model = “t1”) requires more prior estimations than $\mathcal {M}_{\text {PBK}}$ (model = “t2”) and $\mathcal {M}_{\text {NB}}$ (model = p1), due to use of epigenetic information. Depending on the type of model parameter fed to the function dataStorage, the probabilities for the following factors are estimated:

1. Repressive histone mark *H*3*K*27*m* *e*3 for *D* *A* *C* *T*3 11 loci from [2] was adopted. Via fold enrichment, the effects of the *H*3*K*27*m* *e*3 were found 500 bp downstream of and near the *D* *A* *C* *T*3 transcription start site (TSS) in HT29 cells. These marks were recorded via chromatin immuno-precipitation (ChiP) assays and enriched at 11 different loci in the 3.5- to 3.5-kb region of the DACT3 TSS. Fold enrichment measurements of *H*3*K*27*m* *e*3 for normal *F* *H* *s*74*I* *n* *t* and cancerous *S* *W*480 were recorded and normalized. The final probabilities are the average of these normalized values of enrichment measurements.
2. Active histone mark *H*3*K*4*m* *e*3 for *D* *A* *C* *T*3 loci from [2] was adopted. Via fold enrichment, the effects of the *H*3*K* *m* *e*3 were found 500 bp downstream of and near the *D* *A* *C* *T*3 transcription start site (TSS) in HT29 cells. These marks were recorded via chromatin immuno-precipitation (ChiP) assays and enriched at 11 different loci in the 3.5- to 3.5-kb region of the *D* *A* *C* *T*3 TSS. Fold enrichment measurements of *H*3*K*4*m* *e*3 for normal *F* *H* *s*74*I* *n* *t* and cancerous *S* *W*480 were recorded and normalized. The final probabilities are the average of these normalized values of enrichment measurements.
3. Fractions for methylation of *D* *K* *K*1 and *W* *I* *F*1 gene taken from [5] via manual counting through visual inspection of intensity levels from methylation-specific PCR (MSP) analysis of gene promoter region and later normalized. These normalized values form the probability estimates for methylation.
4. Fractions for methylation and non-methylation status of *S* *F* *R* *P*1, *S* *F* *R* *P*2, *S* *F* *R* *P*4, and *S* *F* *R* *P*5 (CpG islands around the first exons) was recorded from six affected individuals each having both primary CRC tissues and normal colon mucosa from [6] via manual counting through visual inspection of intensity levels from MSP analysis of gene promoter region and later normalized. These normalized values form the probability estimates for methylation.
5. Methylation of *D* *A* *C* *T*1 (+52 to +375 BGS) and *D* *A* *C* *T*2 (+52 to +375 BGS) in promoter region for *Normal*, *H* *T*29, and *RKO* cell lines from [2] was recorded via counting through visual inspection of open or closed circles indicating methylation status estimated from bisulfite sequencing analysis and later normalized. The averaged values of these normalizations form the probability estimates for methylation.
6. Concentration of *D* *V* *L*2 decreases with expression of *D* *A* *C* *T*3 and vice versa [2]. Due to the lack of exact proportions, the probability values were assumed.
7. Concentration of *β*-*catenin*-given concentrations of *D* *V* *L*2 and *D* *A* *C* *T*1 varies; and for static model, it is tough to assign probability values. High *D* *V* *L*2 concentration or suppression (expression) of *D* *A* *C* *T*1 leads to increase in the concentration of *β*-*catenin* [2, 7]. Wet lab experimental evaluations might reveal the factual proportions.
8. Similarly, the concentrations of *TRCMPLX* [8, 9] and *T* *C* *F*4 [3] have been assumed based on their known roles in the Wnt pathway. Actual proportions as probabilities require further wet lab tests.
9. Finally, the probability of *Sample* being tumorous or normal is a 50 *%* chance level as it contains an equal amount of cancerous and normal cases.

Note that all these probabilities have been recorded in Table 1 of [1] and their values stored in the *probabilities.mat* file.

### Building the Bayesian network model

Next comes the topology of the network using prior biological knowledge which is made available from the results of wet lab experiments documented in literature. This network topology is achieved using the function generateInteraction in the file generateInteraction.m. The function takes in the set of uniqueGenes and the type of the model and generates a cell of interaction for the Bayesian network as well as a cell of unique set of names of the nodes, i.e., Nodenames. A cell is like a matrix but with elements that might be of different types. The indexing of a cell is similar to that of a matrix except for the use of parenthesis instead of square brackets. interaction contains all the prior established biological knowledge that carries causal semantics in the form of arcs between the parent and child nodes. It should be noted that even though the model is not complete due to its static nature, it has the ability to encode prior causal relationships and has the potential for further refinement. Note that a model not being complete does not conclude that the results will be wrong.



The interaction and nodeNames are used as input arguments to the function mk_adj_mat, which then generates an adjacency matrix for a directed acyclic graph (DAG) stored in dag. Using functions biograph and input arguments dag and nodeNames generates a structure gObj that can be used to view the topology of the network. A crude representation of $\mathcal {M}_{\text {PBK+EI}}$ and $\mathcal {M}_{\text {NB+MPBK}}$ shown in Figs. 2 and 4 was generated using the function view. 

Once the adjacency matrix is ready, the initialization of the Bayesian network can be done easily. The total number of nodes is stored in N and the size of the nodes are defined in nodeSizes. In this project, each node has a size of two as they contain discrete values representing binary states. Here, the function ones defines a row vector with N columns. The total number of discrete nodes is defined in discreteNodes. Finally, the Bayesian network is created using the function mk_bnet from the BNT that takes the following as input arguments: (1) dag—the adjacency matrix, (2) nodeSizes—defines the size of the nodes, and (3) discreteNodes—the vector of nodes with their indices marked to be discrete in the Bayesian network and dumps the network in the variable bnet. bnet is of the type STRUCTURE which contains fields, each of which can be of different types like vector, character, array, matrix, cell, or structure. The contents of a field of a structure variable (say bnet), with proper indices, if necessary can be accessed and seen using “bnet.fieldname.” 



### Holdout experiment

After the framework of the Bayesian network has been constructed and initialized, the holdout experiment is conducted. The purpose of conducting the experiment is to generate results on different test data while training the Bayesian network with different sets of training data. From [1], the design of the experiment is a simple 2-holdout experiment where one sample from the normal and one sample from the tumor are paired to form a test dataset. Excluding the pair formed in an iteration of 2-holdout experiment, the remaining samples are considered for training of a BN model. Thus, in a dataset of 24 normal and 24 tumorous cases, an iteration will have a training set which will contain 46 samples and a test set which will contain 2 samples (one of normal and one of tumor). This procedure is repeated for every normal sample which is combined with each of the tumorous sample to form a series of test dataset. In total, there will be 576 pairs of test data and 576 instances of training data. Note that for each test sample in a pair, the expression value for a gene is discretized using a threshold computed for that particular gene from the training set. Computation of threshold will be elucidated later. This computation is repeated for all genes per test sample. Based on the available evidences from the state of expression of all genes that constitute the test data, inference regarding the state of the both *β*-*catenin* transcription complex and the test sample is made. These inferences reveal (a) hidden biological relationship between the expressions of the set of genes under consideration and the *β*-*catenin* transcription complex and (b) information regarding the activation state of the *β*-*catenin* transcription complex and the state of the test sample, as a penultimate step to the proposed hypothesis testing. Two-sample Kolmogorov-Smirnov (KS) test was employed to measure the statistical significance of the distribution of predictions of the states of the previously mentioned two factors.

Apart from testing the statistical significance between the states of factors, it was found that the prediction results for the factors obtained from models including and excluding epigenetic information were also significantly different. The receiver operator curve (ROC) graphs and their respective area under the curve (AUC) values indicate how the predictions on the test data behaved under different models. Ideally, high values of AUC and steepness in ROC curve indicate good quality results.

The holdout experiment begins with the computation of the total number of positive and negative labels present in the whole dataset as well as the search of the indices of the labels. For this, the values in the variable noSamples and transGroundTruthLabels computed from function readCustomFile are used. noPos (noNeg) and posLabelIdx (negLabelIdx) store the number of positive (negative) labels and their indices, respectively.



For storing results as well as the number of times the experiment will run, variables runCnt and Runs are initialized. Runs is of the type structure. The condition in the *if* statement is not useful now and will be described later. 



For each and every positive (cancerous) and negative (normal) labels, the number of times the experiments run is incremented in the count variable runCnt. Next, the indices for test data is separated by using the *i*th positive and the *j*th negative label and these indices are stored in testDataIdx. The test data itself is then separated from expressionMatrix using the testDataIdx and stored in dataForTesting. The corresponding ground truth labels of the test data are extracted from transGroundTruthLabels using testDataIdx and stored in labelForTesting.



After the storage of the test data and its respective indices, trainingDataIdx is used to store the indices of training data by eliminating the indices of the test data. This is done using temporary variables tmpPosLabelIdx and tmpNegLabelIdx. trainingDataIdx is used to store the training data in variable dataForTraining using expressionMatrix and the indices of training data in variable labelForTraining using transGroundTruthLabels. 



#### Defining and estimating probabilities and conditional probabilities tables for nodes in bnet

Till now, the probabilities as well as conditional probability tables (cpt) for some of the nodes have been stored in the *probabilities.mat* file and loaded in the workspace. But the cpt for all the nodes in the bnet remain uninitialized. The *next procedure* is to initialize the tables using assumed values for some of the known nodes while estimating the entries of cpt for other nodes (i.e., of nodes representing genes) using the training data.

To this end, it is important to define a variable by the name cpdStorage of the format structure. Starting with all the nodes that have no parents and whose probabilities and cpt have been loaded in the workspace (saved in *probabilities.mat*), the *for* loop iterates through all the nodes in the network defined by N, stores the index of the *k*th node in nodeidx using function bnet.names with input argument nodeNames{k} and assigns values to cpt depending on the type of the model. If $\mathcal {M}_{\text {PBK+EI}}$ (model = “t1”) is used and the *k*th entry in nodeNames matches with *T* *C* *F*4, then the cpt value in PrTCF4 is assigned to cpt. The parent node of this node is assigned a value 0 and stored in cpdStorage(k).parentnode{1}. The name *T* *C* *F*4 or nodeNames{k} is assigned to cpdStorage(k).node. The cpt values in cpt is assigned to cpdStorage(k).cpt. Finally, the conditional probability density cpt for the node with name *T* *C* *F*4 is stored in bnet.CPD using function tabular_CPD, the Bayesian network bnet, the node index nodeidx, and cpt. Similarly, values in PrMeDKK1, avgPrMeDACT1, avgPrMeDACT2, avgPrH3K27me3, avgPrH3K4me3, PrMeSFRP1, PrMeSFRP2, PrMeSFRP4, PrMeSFRP5, PrMeWIF1, and PrSample initialize the cpt values for nodes *M* *e* *D* *A* *C* *T*1, *M* *e* *D* *A* *C* *T*2, *H*3*k*27*m* *e*3, *H*3*k*4*m* *e*3, *M* *e* *S* *F* *R* *P*1, *M* *e* *S* *F* *R* *P*2, *M* *e* *S* *F* *R* *P*4, *M* *e* *S* *F* *R* *P*5, *M* *e* *W* *I* *F*1, and *Sample*, respectively. It might not be necessary to hard code the variables and more efficient code could be written. Currently, the selection of the hard-coded variables is for ease in reading the code from a biological point of view for person with computer science background. But surely, this programming style is bound to change when large and diverse datasets are employed.

Similar initializations happen for models $\mathcal {M}_{\text {PBK}}$ (model = “t2”) and $\mathcal {M}_{\text {NB+MPBK}}$ (model = “p1”). It should be noted that in $\mathcal {M}_{\text {PBK}}$ ($\mathcal {M}_{\text {NB+MPBK}}$), the only nodes without parents are *T* *C* *F*4 and *Sample* (*T* *C* *F*4 and *BETACAT*). To accommodate for these models, the necessary *elseif* statements have been embedded in the *for* loop below.



In the same *for* loop above, the next step is to initialize probability as well as the cpt values for nodes with parents. Two cases exist in the current scenario, i.e., nodes that (1) represent genes and (2) do not represent genes. To accommodate for gene/non-gene node classification, a logical variable GENE is introduced. Also, before entering the second *for* loop described below, a variable gene_cpd of the format structure is defined for storage of the to be computed cpt values for all genes in the dataset. parentidx stores the indices of the parents of the child node under consideration using the child’s index in nodeidx via bnet.parents{nodeidx}. The total number of parents a child node has is contained in noParents.

Initially, GENE is assigned a value of 0 indicating that the node under consideration is not a gene node. If this is the case, the ˜GENE in the *if* condition of the *for* loop below gets executed. In this case, depending on the type of the model cpt values of a particular node is initialized. For $\mathcal {M}_{\text {PBK+EI}}$ and $\mathcal {M}_{\text {PBK}}$ (model = “t1” and model = “t2”), the cpt values for nodes *BETACAT*, *D* *V* *L*2, and *TRCMPLX* is stored using values in PrBETACAT, PrDVL2, and PrTRCMPLX. As before, using the function tabular_CPD and values in nodeidx, bnet, and cpt as input arguments, the respective cpt is initialized in bnet.CPD{nodeidx}. Similar computations are done for $\mathcal {M}_{\text {NB+PBK}}$, i.e., model “p1” for node TRCMPLX. Finally, the indices of the parents of the *k*th child node are stored in cpdStorage(k).parentnode{m}.

On the other hand, if the name of the node in the *k*th index of nodeNames matches the name in the *l*th index of uniqueGenes, a parent variable of format cell is defined within the second nested *for* loop below. The names of the parents are stored in this variable using nodeNames{parentidx(n)}. Next, the cpt values of these parent nodes are separately stored using a cell parent_cpd and a count cnt. Finally, the cpd values for the *l*th gene is determined using the function generateGenecpd in the script *generateGenecpd.m* that takes the following input arguments: (1) vecTraining—gene expression from training data, (2) labelTraining—labels for training data, (3) nodeName—name of the gene involved, (4) parent—name of parents of the child node or the gene under consideration, (5) parent_cpd—parent cpd values, (6) model—kind of model and finally returns the output as a structure gene_cpd containing cpd for the particular gene under consideration given its parents as well as a threshold value in the form of median. In the code below, the values of the following variables are used as input arguments for the function generateGenecpd, in order: (1) dataForTraining(l,:)—training data for the *l*th unique gene, (2) labelForTraining—labels for the training data, (3) uniqueGenes{l}, (4) parent, (5) parent_cpd, (6) model. The output of the function is stored in the structure variable x. The threshold at which the probabilities were computed for the *l*th gene is stored in gene_cpd(l).vecmedian using x.vecmedian and the probabilities themselves are stored in gene_cpd(l).T using x.T. These probabilities are reshaped into a row vector and stored in cpt. As mentioned before, using function tabular_CPD and values in nodeidx, bnet and cpt as input arguments, the respective cpt is initialized in bnet.CPD{nodeidx}. Finally, the required values of cpt, name of *l*th gene or *k*th node and indices of its parent nodes are stored in cpdStorage(k).cpt, cpdStorage(k).node and cpdStorage(k).parentnode{m}, respectively.

It should be noted that the exposition of the generation of probability values for the different genes via the function generateGenecpd needs a separate treatment and will be addressed later. To maintain the continuity of the workflow of the program, the next step is addressed after the code below.



#### Evidence building and inference

The values estimated in gene_cpd as well as cpdStorage are stored for each and every run of the holdout experiment. Also, the dimensions of the testing data are stored. 



Next, depending on the type of the evidence provided in eviDence, inferences can be made. Below, a section of code for the gene expression evidence, which gets executed when the *case* “ge” matches with the parameter eviDence of the *switch* command, is explained. The issue that was to be investigated was whether the *β*-*catenin*-based *TRCMPLX* is always switched on (off) or not when the *Sample* is cancerous (normal). In order to analyze this biological issue from a computational perspective, it would be necessary to observe the behavior of the predicted states of both *TRCMPLX* as well as *Sample*, given all the available evidence. For this purpose, the variable tempTRCMPLXgivenAllge is defined as a vector for each model separately, while the variable tempSAMPLE is defined as a vector for biologically inspired models, i.e., $\mathcal {M}_{\text {PBK+EI}}$ and $\mathcal {M}_{\text {PBK}}$ separately. This is due to the assumption that the state of *TRCMPLX* is the same as the state of the test sample under consideration in the $\mathcal {M}_{\text {NB+MPBK}}$ (a modification of [3]).

In the section of the code below, *for* each of the test dataset, an evidence variable of the format cell is defined. The evidence is of the size equivalent to the number of node N in the network. Only those indices in the cell will be filled for which information is available from the test data. Since the function twoHoldOutExp started with “ge” as an argument for the type of evidence, evidence will be constructed from information available via gene expression from the test data. Thus for the *m*th gene, if the gene expression in the test data (i.e., dataForTesting(m,k)) is lower than the threshold generated using the median of expressions for this gene in the training data (i.e., gene_cpd(m).vecmedian), then the evidence for this gene is considered as inactive or repressed, i.e., evidence{bnet.names(uniqueGenes(m))} = 1, else the evidence for this gene is considered as active or expressed, i.e., evidence{bnet.names (uniqueGenes(m))} = 2. Iterating through all the genes, the evidence is initialized with the available information for the *k*th test data.

Once the probability values have been initialized either by computation or assumption, then for the *k*th test data, a Bayesian network engine is generated and stored in bnetEngine via the junction tree algorithm implemented in function jtree_inf_engine that uses the input argument as the newly initialized network stored in bnet. The bnetEngine is then fed with the values in evidence to generate a new engine that contains the updated probability values for nodes for which there is no evidence in the network. This is done using the function enter_evidence. According to BNT provided by [4], in the case of the jtree engine, enter_evidence implements a two-pass message-passing scheme. The first return argument (engine) contains the modified engine, which incorporates the evidence. The second return argument (loglik) contains the log-likelihood of the evidence. It is the first returned argument or the modified engine that will be of use further. It is important to note that for every iteration that points to a new test data in the *for* loop, a new Bayesian network engine is generated and stored in bnetEngine. If this is not done, then the phenomena of *explaining away* can occur on feeding new evidence to an already modified engine which incorporated the evidence from the previous test data. In *explaining away*, the entering of new evidence might outweigh the effect of an existing influencing factor or evidence thus making the old evidence redundant. This simulation is not related to such study of explaining away.

The belief that the *TRCMPLX* is switched on given the gene expression evidence, i.e., *P* *r*(*T* *R* *C* *M* *P* *L* *X*=2|ge as evidence) is computed by estimating the marginal probability values using the function marginal_nodes which takes the engine stored in engine and the name of the node using bnet.names(’TRCMPLX’). The marginal probabilities are stored in margTRCMPLX. The final probability of *TRCMPLX* being switched on given all gene expression evidences is stored in tempTRCMPLX givenAllge using margTRCMPLX.T(2). Similarly, for biologically inspired models the belief that the test *Sample* is cancerous given the gene expression evidence, i.e., *P* *r*(*S* *a* *m* *p* *l* *e*=2|ge as evidence) is computed using function marginal_nodes that takes the engine stored in engine and the name of the node using bnet.names(’Sample’). The marginal probabilities are stored in margSAMPLE. The final probability of *Sample* being cancerous given all gene expression evidences is stored in tempSAMPLE using margSAMPLE.T(2).



Finally, for a particular count of the run of the experiment, tempTRCMPLXgivenAllge and tempSAMPLE are stored in the structure Runs using different variables associated with Runs. This iteration keeps happening until the 2-holdout experiment is exhausted. The case when eviDence is “me” or evidence for methylation will be discussed later as a programming project.



### Storing results, plotting graphs, and saving files

The final section of the code deals with the storing of the results, plotting of graphs, and saving the results in the files. Since the current explanation is for gene expression evidence, the code pertaining to “ge” is explained. Readers might want to develop the code for evidence regarding methylation as a programming project.

To store results as well as the conditional probabilities for *TRCMPLX* and *Sample* given all the gene expression evidence, a cell variable Results, a counter cntResult, and vector variables condPrTRCMPLXgivenAllge, condPrSAMPLE, and labels are defined as well as initialized. Next, the prediction values and original labels are stored while iterating through the total number of runs of the experiment. This is done using the *for* loop and the variable runCnt. For the *i*th run, predicted conditional probabilities of *TRCMPLX* and *Sample* from each run are stored in condPrTRCMPLXgivenAllge(i,:) and condPrSAMPLE(i,:), depending on the model used. Finally, the ground truth labels of the test data are stored in a matrix where the *i*th row is initialized with labels(i,:) = [-1, +1];. Here, labels in a matrix and −1 (+1) represent normal (cancerous) cases. Next, the variables condPrTRCMPLXgivenAllge and condPrSAMPLE are reshaped into vectors for further processing.

The plotting of the ROC curves and the estimation of their respective AUCs is achieved using function perfcurve that takes labels and either of the vectors condPrTRCMPLXgivenAllge or condPrSAMPLE depending on the type of the model selected. The function churns out useful information in the form of the false positive rate in X, the true positive rate in Y, and the estimated AUC for ROC of condPrTRCMPLXgivenAllge (condPrSAMPLE) in AUCTRCMPLXgivenAllge (AUCSAMPLE). The plot function is used to draw the graphs along with the depiction of legends using function legend. Finally, the two-sample Kolmogorov-Smirnov test between the predictions of states of *TRCMPLX* and *Sample* is performed using the kstest2 function. This function takes the two vectors condPrTRCMPLXgivenAllge and condPrSAMPLE as arguments, compares the distribution of the predictions, and returns the state of significance between the two distributions in h01. If the value of h01 is 1, then statistical significance exists else it does not exist. Sinha [1] shows that the statistical difference exists between predictions of *TRCMPLX* and *Sample* when the nodes for the same are segregated in the biologically inspired causal models, which is not the case with the Naive Bayes model.

Lastly, the computed variables are stored in a.mat file using the function save. Options for using the save function can be obtained from the help command in MATLAB.



The ROC graphs and their respective AUC values found in the figures of [1] are plotted by making variation in the assumed probability values of PrTRCMPLX in the function generateGenecpd. The details of the generateGenecpd are discussed in the next section.

The variation in the assumed probability values of the *TRCMPLX* that affect the behavior of the gene nodes is termed as ETGN in [1]. Since the entire code runs only once, it has to be run for different instances of input arguments, separately. Once the results have been saved in *Results.mat* file, one can rename the file based on the model and the evidence arguments used in function twoHoldOutExp. Thus, if the code is run for model “t1” and ETGN of 90 *%*, then the user needs to rename the *Results.mat* that stores the results with an appropriate file name like *Results-T1-GE-pforTRCMPLX-90per.mat*. Once the results for all permutations of instances for a vector of input arguments in twoHoldOutExp have been obtained, the script geneTRCMPLXstats using the generated.mat result files can be executed to generate the tables which shows how *TRCMPLX* behaves as the evidences of genes vary in both normal and tumorous cases. Tables 5 and 6 in [1] are generated using this script. How interpretations of the results are made can be studied in more depth in the results section of [1]. However, succinctly, the script geneTRCMPLXstats generates mean/average estimates of the conditional probability that the transcription complex will be switched on or off in normal or tumor test samples, given the different gene evidences. By majority, if a gene expression is found to be repressed (active) in normal or tumor case, then the predicted belief represented by the probability of the transcription complex conditional on repression (activation) is chosen as the inferred biological phenomena. Figures 6 and 7 of [1] depict the summarized pictorial representation of the predicted inferences shown in Tables 5 and 6 of [1].

**Fig. 5:**
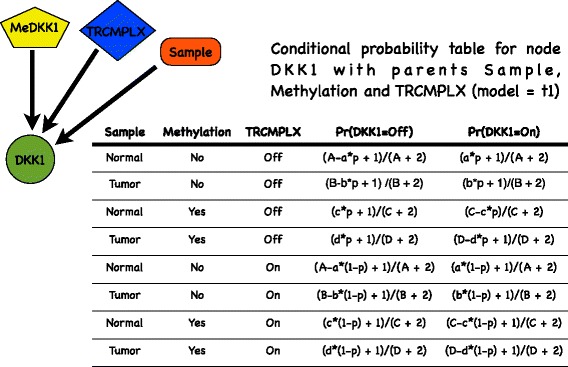
Conditional probability table for node *D* *K* *K*1 in $\mathcal {M}_{PBK+EI}$

Note that to generate the ROC graphs and their respective AUC values for different models with varying effect of *TRCMPLX* on different genes (ETGN in [1]), the results in variables X and Y (of twoHoldOutExp) are stored in different variables and clumped together in a.mat file titled *aucANDpredictions_sample_TRCMPLX.mat*. This has to be done manually for each model and every setting of ETGN. For example, using model t1 and ETGN of 60 *%*, the false positive rate in X is stored as xT1_60 and the true positive rate in Y is stored as yT1_60, in the abovementioned.mat file. Finally, the script in the *m* file titled plotAUC is used to manipulate the aforementioned transformed variables and generate the ROC curves in Figure 5 of the results section of [1]. The Google drive https://drive.google.com/folderview?id=0B7Kkv8wlhPU-T05wTTNodWNydjA707usp=sharing contains the results under the compressed directory with name *Results-2013*. Inference interpretations of the results can be studied in more depth from [1].

Finally, a full section is dedicated to the computation of the probabilities for nodes with parents which has been implemented in function generateGenecpd. The computation of gene nodes happens within the holdout experiment and before the new computation of CPTs conditional on the provided evidence. Since the details of computation of CPTs for gene nodes is dense, it has been treated separately after the explanation of the code of holdout experiment.

### Generating probabilities for gene nodes with parents

Here, the code for the function generateGenecpd is explained. As a recapitulation, the function generateGenecpd in the script *generateGenecpd.m* takes the following input arguments: (1) vecTraining—gene expression from the training data, (2) labelTraining—labels of the training data, (3) nodeName—name of the gene involved, (4) parent—name of parents of the child node or the gene under consideration, (5) parent_cpd—parent cpd values, (6) model—kind of the model and finally returns the output as a structure gene_cpd containing cpd for the particular gene under consideration given its parents as well as a threshold value in the form of median. In the code below, the values of the following variables are used as input arguments for the function generateGenecpd, in order: (1) dataForTraining(l,:)—training data for the *l*th unique gene, (2) labelForTraining—labels for the training data, (3) uniqueGenes{l}, (4) parent, (5) parent_cpd, (6) model. The output of the function is stored in the structure variable x. The threshold at which the probabilities were computed for the *l*th gene is stored in gene_cpd(l).vecmedian using x.vecmedian and the probabilities themselves are stored in gene_cpd(l).T using x.T.

The code begins with the storing of the dimension of a gene expression vector in vecTraining in variables r and c and recording the length of the vector containing the labels of the training data (in labelTraining) in variable lencond. Finally, the much reported threshold is estimated here using the median of the training data and stored in vecmedian.



In [1], the effect of *TRCMPLX* on the gene expression has been analyzed as it is not known to what degree the *TRCMPLX* plays a role in the Wnt signaling pathway. To investigate this, Sinha [1] incorporated a parameter p that encodes the effect of *TRCMPLX* on the expression of the gene which is influenced by it. Thus, while iterating through the list of parents if one encounters *TRCMPLX* as a parent, then p is initialized to a certain value. In [1], the effect of *TRCMPLX* being active (1−*p*) is incremented in steps of 0.1 from {0.5 to 0.9} and respective ROC graphs are plotted using the same.



It is important to note that the computation of gene probabilities differ from model to model and a detailed description of each computation is given for each gene for all three models, before explaining the computation for another gene. Also, from [1], theoretically, for a gene *g*_*i*_∀*i* genes, let there be *n*_tr_ different instances of expression values from the sample training data. Let each of the *n*_tr_ gene expression values be discretized to 0 and 1 based on their evaluation with respect to the median threshold. The 1’s represent the total number of expression where the gene is active and 0’s represent the total number of expression where the gene is inactive. In case of normal and tumorous samples, the proportions of 1’s and 0’s may be different. The median of the expression values is employed as a threshold to decide the frequency of *g*_*i*_ being active or inactive given the state of the parent node(s). This median is also used along with the labels of the training data to decide the status of different parent factors affecting the gene under consideration.

If one observes the network in Figs. 2 and 4, one finds that there are nodes that have one, two, or three parent nodes. Computation of conditional probability tables for these child nodes which represent gene expression for both tumor and normal samples in the different models (i.e., “t1” for $\mathcal {M}_{\text {PBK+EI}}$, “t2” for $\mathcal {M}_{\text {PBK}}$, and “p1” for $\mathcal {M}_{\text {NB+MPBK}}$) require intuitive analysis of the expression data. Estimation of the cpts for three gene nodes, i.e., *D* *K* *K*1, *D* *K* *K*2, and *D* *A* *C* *T*3, each having different parents depending on the type of the model has been explained below. Nodes that have similar corresponding behavior are enlisted but the estimation is not derived.

#### DKK1 in $\mathcal {M}_{\text {PBK+EI}}$ (t1)

Since there are three parents for *D* *K* *K*1, namely *M* *e* *D* *K* *K*1, *Sample*, and *TRCMPLX*, the cpt values for the table is segregated based on the status of methylation and quality of samples. A 2×2 cross table for methylation and sample generates frequency estimates that can help derive probability values. The entries of the cross table depict the following cases: (a) methylated in normal (represented by vector mINn), (b) un-methylated in normal (represented by vector umINn), (c) methylated in tumorous (represented by vector mINt), and (d) un-methylated in tumorous (represented by vector umINt) cases. For every *j*th entry in the vecTraining, if the label (labelTraining(j)) is normal (≤0) and the *D* *K* *K*1 gene expression (vecTraining(j)) is less than the estimated median (≤vecmedian), then the value in vecTraining(j) is appended to mINn. Here, expression level lower than median indicates probable repression due to methylation in normal case. If the label (labelTraining(j)) is normal (≤0) and the *D* *K* *K*1 gene expression (vecTraining(j)) is greater than the estimated median (≥vecmedian), then the value in vecTraining(j) is appended to umINn. Here, expression level greater than median indicates probable activation due to un-methylation in normal case. If the label (labelTraining(j)) is tumorous (≥0) and the *D* *K* *K*1 gene expression (vecTraining(j)) is less than the estimated median (≤vecmedian), then the value in vecTraining(j) is appended to mINt. Here, expression level lower than median indicates probable repression due to methylation in tumorous case. And finally, if the label (labelTraining(j)) is tumorous (≥0) and the *D* *K* *K*1 gene expression (vecTraining(j)) is greater than the estimated median (≥vecmedian), then the value in vecTraining(j) is appended to umINt. Here, expression level greater than median indicates probable activation due to un-methylation in tumorous case.



Also, since the actual probability values for the activation of the *TRCMPLX* is not known, the conditional probabilities are multiplied with a probability value of *p* when the *TRCMPLX* is off and with a probability value 1−*p* when the *TRCMPLX* is on. Before estimating the values for cpt of *D* *K* *K*1, it is important to see how (1) the probability table would look like and (2) the probability table is stored in BNT [4]. Table 10 represents the conditions of sample as well as the methylation along with transcription complex and the probable beliefs of events (*D* *K* *K*1 being on/off). With three parents and binary state, the total number of conditions is 2^3^. To estimate the values of the probable beliefs of an event, the following computation is done. (*Case* - *TRCMPLX* is Off) The Pr(*D* *K* *K*1 - On |*S* *a* *m* *p* *l* *e* - Normal, *Me* - UM) being low is the fraction of number of 1’s in the normal sample (a ×*p*) and the sum of total number of normal samples and number of 1’s in the tumorous samples, i.e., the non-methylated gene expression values in tumorous samples (A). Similarly, Pr(*D* *K* *K*1 - On |*S* *a* *m* *p* *l* *e* - Tumor, *Me* - UM) being low is the fraction of number of 1’s in the tumorous sample (b ×*p*) and the sum of total number of tumorous samples and number of 1’s in the normal samples, i.e., the non-methylated gene expression values in normal samples (B). Again, Pr(*D* *K* *K*1 - Off |*S* *a* *m* *p* *l* *e* - Normal, *Me* - M) being high is the fraction of number of 0’s in the normal sample (c ×*p*) and the sum of total number of normal samples and number of 0’s in the tumorous samples, i.e., the methylated gene expression values in tumorous samples (C). Finally, Pr(*D* *K* *K*1 - Off |*S* *a* *m* *p* *l* *e* - Tumor, *Me* - M) being high is the fraction of number of 0’s in the tumorous sample (d ×*p*) and the sum of total number of tumorous samples and number of 0’s in the normal samples, i.e the methylated gene expression values in normal samples (D).

**Table 10:** Conditional probability table for *D* *K* *K*1 in $\mathcal {M}_{\text {PBK+EI}}$ (model - t1)

CPT for *D* *K* *K*1 in $\mathcal {M}_{\text {PBK+EI}}$(model - t1)				
*Sample*	*Methylation*	*TRCMPLX*	Pr(*D* *K* *K*1=Off)	Pr(*D* *K* *K*1=On)
Normal	No	Off	h (1)	l (9)
Tumor	No	Off	h/l (2)	l/h (10)
Normal	Yes	Off	h (3)	l (11)
Tumor	Yes	Off	h (4)	l (12)
Normal	No	On	h (5)	l (13)
Tumor	No	On	h/l (6)	l/h (14)
Normal	Yes	On	h (7)	l (15)
Tumor	Yes	On	h (8)	l (16)

h - probability of event being high; l - probability of event being low. Serial numbers in brackets represent the ordering of numbers in vectorial format

(*Case* - *TRCMPLX* is On) Next, the Pr(*D* *K* *K*1 - On |*S* *a* *m* *p* *l* *e* - Normal, *Me* - UM) being low is the fraction of number of 1’s in the normal sample (a ×(1−*p*)) and the sum of total number of normal samples and number of 1’s in the tumorous samples, i.e., the non-methylated gene expression values in tumorous samples (A). Similarly, Pr(*D* *K* *K*1 - On |*S* *a* *m* *p* *l* *e* - Tumor, *Me* - UM) being low is the fraction of number of 1’s in the tumorous sample (b ×(1−*p*)) and the sum of total number of tumorous samples and number of 1’s in the normal samples, i.e., the non-methylated gene expression values in normal samples (B). Again, Pr(*D* *K* *K*1 - Off |*S* *a* *m* *p* *l* *e* - Normal, *Me* - M) being high is the fraction of number of 0’s in the normal sample (c ×(1−*p*)) and the sum of total number of normal samples and number of 0’s in the tumorous samples, i.e., the methylated gene expression values in tumorous samples (C). Finally, Pr(*D* *K* *K*1 - Off |*S* *a* *m* *p* *l* *e* - Tumor, *Me* - M) being high is the fraction of number of 0’s in the tumorous sample (d ×(1−*p*)) and the sum of total number of tumorous samples and number of 0’s in the normal samples, i.e., the methylated gene expression values in normal samples (D). Complementary conditional probability values for *D* *K* *K*1 being inactive can easily be computed from the above estimated values.



These values are stored in variable T and the estimation is shown in the following section of the code. After the values in T have been established, a constant 1 is added as pseudo-count to convert the distribution to a probability distribution via Dirichlet process. This is done to remove any deterministic 0/1 values appearing in the probability tables. If 0/1 appears in the probability tables then one has deterministic evidence regarding an event and the building of the Bayesian engine collapses. These counts also represent the unobserved that might not have been recorded due to small sample size. The Dirichlet process is a generalization of the Dirichlet distribution which is parameterized by a vector of positive reals. The pseudo-counts here form the positive values. What this basically means is that the probability density function returns the belief that the probabilities of some rival events given that each event has been observed non-negative number of times. These distributions are often used as prior distributions in Bayesian statistics.

Finally, the frequencies/probabilities in T are normalized in order to obtain the final conditional probability values for *D* *K* *K*1. Estimation of cpts for genes *S* *F* *R* *P*1, *W* *I* *F*1 and *D* *K* *K*4 which have methylation, *TRCMPLX* and *Sample* as parents require same computations as above. Figure 5 shows the pictorial representation of one of the cpt in $\mathcal {M}_{\text {PBK+EI}}$.

#### DKK1 in $\mathcal {M}_{\text {PBK}}$ (t2)

There are two parents for *D* *K* *K*1, namely *TRCMPLX* and *Sample*. The conditional probability value for a gene being active or inactive is estimated based on the state of the *Sample*. Again, since the actual probability values for the activation of the *TRCMPLX* is not known the conditional probabilities are multiplied with a probability value of *p* when the *TRCMPLX* is off and with probability value 1−*p* when the *TRCMPLX* is on.

The analysis of quality of sample generates frequency estimates that can help derive probability values. These frequencies depict the following cases: (a) gene repressed in normal (represented by vector offINn), (b) gene expressed in normal (represented by vector onINn), (c) gene repressed in tumorous (represented by vector offINt), and (d) gene expressed in tumorous (represented by vector onINt) cases. For every *j*th entry in the vecTraining, if the label (labelTraining(j)) is normal (≤0) and the *D* *K* *K*1 gene expression (vecTraining(j)) is less than the estimated median (≤vecmedian), then the value in vecTraining(j) is appended to offINn. Here, expression level lower than median indicates probable gene repression in the normal case. If the label (labelTraining(j)) is normal (≤0) and the *D* *K* *K*1 gene expression (vecTraining(j)) is greater than the estimated median (≥vecmedian), then the value in vecTraining(j) is appended to onINn. Here, expression level greater than median indicates probable gene activation in normal case. If the label (labelTraining(j)) is tumorous (≥0) and the *D* *K* *K*1 gene expression (vecTraining(j)) is less than the estimated median (≤vecmedian), then the value in vecTraining(j) is appended to offINt. Here, expression level lower than median indicates probable gene repression in tumor case. And finally, If the label (labelTraining(j)) is tumorous (≥0) and the *D* *K* *K*1 gene expression (vecTraining(j)) is greater than the estimated median (≥vecmedian), then the value in vecTraining(j) is appended to onINt. Here, expression level greater than median indicates probable gene activation in tumorous case.



Again, before estimating the values for cpt of *D* *K* *K*1, it is important to see how (1) the probability table would look like and (2) the probability table is stored in BNT [4]. Table 11 represents the conditions of *Sample* as well as *TRCMPLX* and the probable beliefs of events (*D* *K* *K*1 being on/off). With two parents and binary state, the total number of conditions is 2^2^. To estimate the values of the probable beliefs of an event, the following computation is done. The probability of gene expression being active given *Sample* is normal and *TRCMPLX* is off, i.e., Pr(*D* *K* *K*1 = Active |*S* *a* *m* *p* *l* *e* = Normal, *TRCMPLX* = Off), is the fraction of number of 1’s in the normal sample (a ×*p*) and the sum of total number of normal samples (A). Similarly, the probability of gene expression being active given *Sample* is tumorous and *TRCMPLX* is off, i.e., Pr(*D* *K* *K*1 = active |*S* *a* *m* *p* *l* *e* = tumorous, *TRCMPLX* = Off), is the fraction of number of 1’s in the tumorous sample (b ×*p*) and the sum of total number of tumorous samples (B). Again, the probability of gene expression being inactive given *Sample* is normal and *TRCMPLX* is on, i.e., Pr(*D* *K* *K*1 = inactive |*S* *a* *m* *p* *l* *e* = normal, *TRCMPLX* = On), is the fraction of number of 0’s in the normal sample (A-a ×(1−*p*)) and the sum of total number of normal samples (A). Lastly, the probability of gene expression being inactive given *Sample* is tumorous and *TRCMPLX* is on, i.e., Pr(*D* *K* *K*1 = inactive |*S* *a* *m* *p* *l* *e* = tumorous, *TRCMPLX* = On), is the fraction of number of 0’s in the tumorous sample (B-b ×(1−*p*)) and the sum of total number of tumorous samples (b). Complementary conditional probability values for *D* *K* *K*1 being inactive can easily be computed from the above estimated values.

**Table 11:** Conditional probability table for *D* *K* *K*1 in $\mathcal {M}_{\text {PBK}}$ (model - t2)

CPT for *D* *K* *K*1 in $\mathcal {M}_{\text {PBK}}$ (model - t2)			
*Sample*	*TRCMPLX*	Pr(*D* *K* *K*1=Off)	Pr(*D* *K* *K*1=On)
Normal	Off	h (1)	l (5)
Tumorous	Off	l (2)	h (6)
Normal	On	h (3)	l (7)
Tumorous	On	l (4)	h (8)

h - probability of event being high; l - probability of event being low. Serial numbers in brackets represent the ordering of numbers in vectorial format

**Fig. 6:**
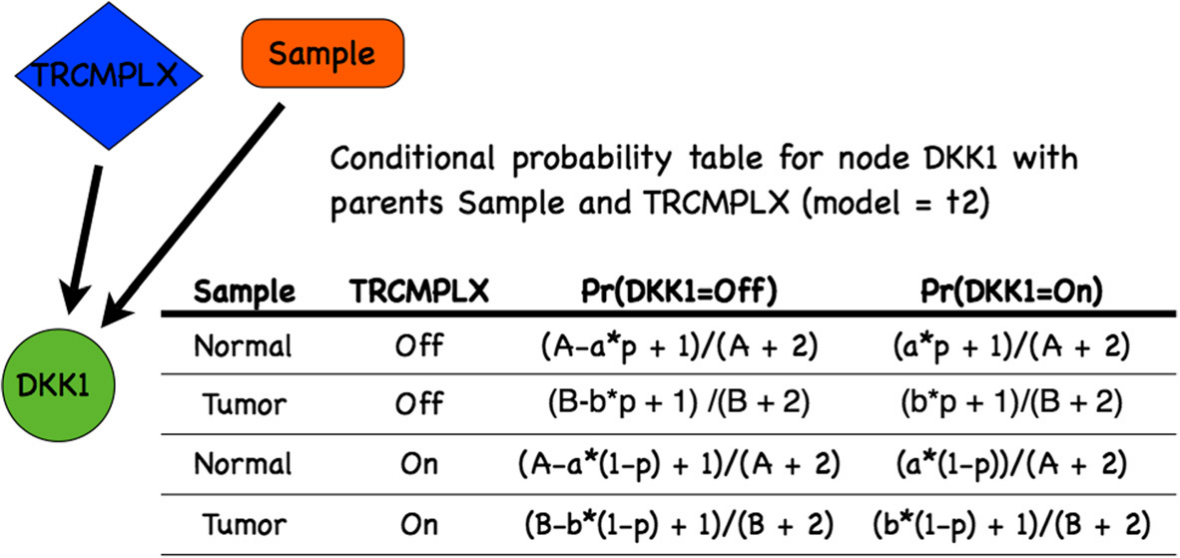
Conditional probability table for node *D* *K* *K*1 in $\mathcal {M}_{PBK}$

After the values in T have been established, a constant 1 is added as pseudo-count to convert the distribution to a probability distribution via Dirichlet process. Finally, the frequencies in T are normalized in order to obtain the final conditional probability values for *D* *K* *K*1. Estimation of cpts for genes *S* *F* *R* *P*1, *C* *C* *N* *D*1, *C* *D*44, *W* *I* *F*1, *MYC*, and *D* *K* *K*4 which has *TRCMPLX* and *Sample* as parents requires the same computations as above. Figure 6 shows the pictorial representation of one of the cpt in $\mathcal {M}_{\ \text {PBK}}$.

#### DKK1 in $\mathcal {M}_{\text {NB+MPBK}}$ (p1)

Following the naive Bayes model presented by [3] and making slight modifications to it, Sinha [1] generated $\mathcal {M}_{\text {NB+MPBK}}$. In this, all genes have a single parent, namely *TRCMPLX*, and it is assumed that the predicted state of *TRCMPLX* is exactly the same as the quality of the test sample. Thus, the initial probability values for *TRCMPLX* are assumed to be fixed and no variation is made on it. The conditional probability value for a gene being active or inactive is estimated based on the state of the *TRCMPLX*.

The segregation of the probability values depends on the following conditions: (a) gene is active and *TRCMPLX* is on (represented by vector onINTrOn), (b) gene is inactive and *TRCMPLX* is off (represented by vector offINTrOn), (c) gene is active and *TRCMPLX* is off (represented by vector onINTrOff), and (d) gene is inactive (represented by vector offINTrOff). For every *j*th entry in the vecTraining, if the label (labelTraining(j)) is ≤0 (*TRCMPLX* is off) and the *D* *K* *K*1 gene expression (vecTraining(j)) is less than the estimated median (≤vecmedian), then the value in vecTraining(j) is appended to offINTrOff. If the label (labelTraining(j)) is ≤0 (*TRCMPLX* is off) and the *D* *K* *K*1 gene expression (vecTraining(j)) is greater than the estimated median (≥vecmedian), then the value in vecTraining(j) is appended to onINTrOff. If the label (labelTraining(j)) is ≥0 (*TRCMPLX* is on) and the *D* *K* *K*1 gene expression (vecTraining(j)) is less than the estimated median (≤vecmedian), then the value in vecTraining(j) is appended to offINTrOn. And finally, if the label (labelTraining(j)) is ≥0 (*TRCMPLX* is on) and the *D* *K* *K*1 gene expression (vecTraining(j)) is greater than the estimated median (≥vecmedian), then the value in vecTraining(j) is appended to onINTrOn.



Lets again see how (1) the probability table would look like and (2) the probability table is stored in BNT [4] before estimating the values for cpt of *D* *K* *K*1. Table 12 represents the conditions of *TRCMPLX* and the probable beliefs of events (*D* *K* *K*1 being on/off). With a single parent and binary state, the total number of conditions is 2^1^. To estimate the values of the probable beliefs of an event, the following computation is done. The probability of gene expression being active given *TRCMPLX* is off, i.e., Pr(*D* *K* *K*1 = Active |*T* *R* *C* *M* *P* *L* *X* = Off), is the fraction of number of 1’s in the normal sample (a) and the sum of total number of normal samples (A). Similarly, the probability of gene expression being inactive given *TRCMPLX* is off, i.e., Pr(*D* *K* *K*1 = active |*T* *R* *C* *M* *P* *L* *X* = On), is the fraction of number of 1’s in the tumorous sample (b) and the sum of total number of tumorous samples (B). Complementary conditional probability values for *D* *K* *K*1 being inactive can easily be computed from the above estimated values. Figure 6 shows the pictorial representation of one of the cpt in $\mathcal {M}_{\text {PBK}}$.

**Table 12:** Conditional probability table for *D* *K* *K*1 in $\mathcal {M}_{\text {NB+MPBK}}$ (model - p1)

CPT for *D* *K* *K*1 in $\mathcal {M}_{\text {NB+PBK}}$ (model - p1)		
*TRCMPLX*	Pr(*D* *K* *K*1=Off)	Pr(*D* *K* *K*1=On)
Off	h (1)	l (3)
On	h (2)	l (4)

h - probability of event being high; l - probability of event being low. Serial numbers in brackets represent the ordering of numbers in vectorial format

**Fig. 7:**
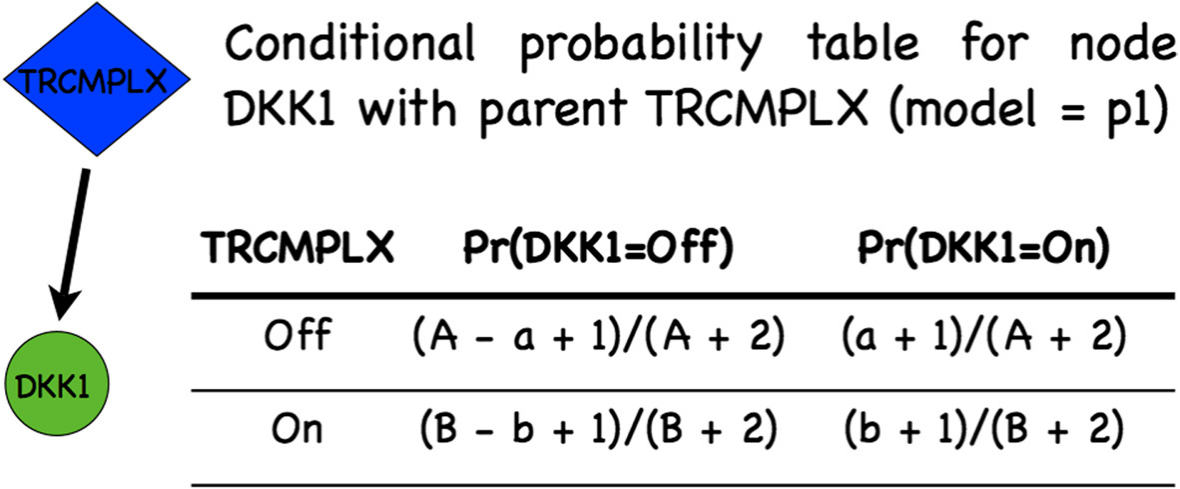
Conditional probability table for node *D* *K* *K*1 in $\mathcal {M}_{\text {NB+MPBK}}$

After the values in T have been established, a constant 1 is added as pseudo count to convert the distribution to a probability distribution via the Dirichlet process. Finally, the frequencies in T are normalized in order to obtain the final conditional probability values for *D* *K* *K*1. Figure 7 shows the pictorial representation of one of the cpt in $\mathcal {M}_{\text {NB+MPBK}}$.

#### DKK2 in $\mathcal {M}_{\text {PBK+EI}}$ (t1)

The *Sample* is the single parent of *D* *K* *K*2. The conditional probability value for a gene being active or inactive is estimated based on the state of the *Sample*. The analysis of quality of sample generates frequency estimates that can help derive probability values. These frequencies depict the following cases: (a) gene repressed in normal (represented by vector offINn), (b) gene expressed in normal (represented by vector onINn), (c) gene repressed in tumorous (represented by vector offINt), and (d) gene expressed in tumorous (represented by vector onINt) cases. For every *j*th entry in the vecTraining, if the label (labelTraining(j)) is normal (≤0) and the *D* *K* *K*2 gene expression (vecTraining(j)) is less than the estimated median (≤vecmedian), then the value in vecTraining(j) is appended to offINn. Here, expression level lower than median indicates probable gene repression in the normal case. If the label (labelTraining(j)) is normal (≤0) and the *D* *K* *K*2 gene expression (vecTraining(j)) is greater than the estimated median (≥vecmedian), then the value in vecTraining(j) is appended to onINn. Here, expression level greater than median indicates probable gene activation in the normal case. If the label (labelTraining(j)) is tumorous (≥0) and the *D* *K* *K*2 gene expression (vecTraining(j)) is less than the estimated median (≤vecmedian), then the value in vecTraining(j) is appended to offINt. Here, expression level lower than median indicates probable gene repression in the tumor case. And finally, If the label (labelTraining(j)) is tumorous (≥0) and the *D* *K* *K*2 gene expression (vecTraining(j)) is greater than the estimated median (≥vecmedian), then the value in vecTraining(j) is appended to onINt. Here, expression level greater than median indicates probable gene activation in the tumorous case.



Lets again see how (1) the probability table would look like and (2) the probability table is stored in BNT [4] before estimating the values for cpt of *D* *K* *K*2. Table 13 represents the conditions of *Sample* and the probable beliefs of events (*D* *K* *K*2 being on/off). With a single parent and binary state, the total number of conditions is 2^1^. To estimate the values of the probable beliefs of an event, the following computation is done. The probability of gene expression being active given *Sample* is normal, i.e., Pr(*D* *K* *K*2 = Active |*S* *a* *m* *p* *l* *e* = Normal), is the fraction of number of 1’s in the normal sample (a) and the sum of total number of normal samples (A). Similarly, the probability of gene expression being active given *Sample* is tumorous, i.e., Pr(*D* *K* *K*2 = active |*S* *a* *m* *p* *l* *e* = Tumorous), is the fraction of number of 1’s in the tumorous sample (b) and the sum of total number of tumorous samples (B). Complementary conditional probability values for *D* *K* *K*2 being inactive can easily be computed from the above estimated values.

**Table 13:** Conditional probability table for *D* *K* *K*2 in $\mathcal {M}_{\text {NB+MPBK}}$ (model - t1)

CPT for *D* *K* *K*2 in $\mathcal {M}_{\text {NB+PBK}}$ (model - t1)		
*Sample*	Pr(*D* *K* *K*2=Off)	Pr(*D* *K* *K*2=On)
Normal	l/h (1)	h/l (3)
Tumor	h/l (2)	l/h (4)

h - probability of event being high; l - probability of event being low. Serial numbers in brackets represent the ordering of numbers in vectorial format

After the values in T have been established, a constant 1 is added as pseudo-count to convert the distribution to a probability distribution via Dirichlet process. Finally, the frequencies in T are normalized in order to obtain the final conditional probability values for *D* *K* *K*2. Estimation of cpts for genes *D* *K* *K*3−1, *D* *K* *K*3−2, *S* *F* *R* *P*3, and *L* *E* *F*1 which have *Sample* as parent requires the same computations as above.



#### DKK2 in $\mathcal {M}_{\text {PBK+EI}}$ (t2)

When epigenetic factors are removed from $\mathcal {M}_{\text {PBK+EI}}$ and the model transformed into $\mathcal {M}_{\text {PBK}}$, i.e., model = “t2”, then the estimation of cpt values for *D* *K* *K*2 remain the same as in model = “t1.” The same computations apply for genes *D* *K* *K*3−1, *D* *K* *K*3−2, *S* *F* *R* *P*2, *S* *F* *R* *P*3, *S* *F* *R* *P*4, *S* *F* *R* *P*5, *L* *E* *F*1, *D* *A* *C* *T*1, *D* *A* *C* *T*2, and *D* *A* *C* *T*3, in model = “t2.”

**Fig. 8:**
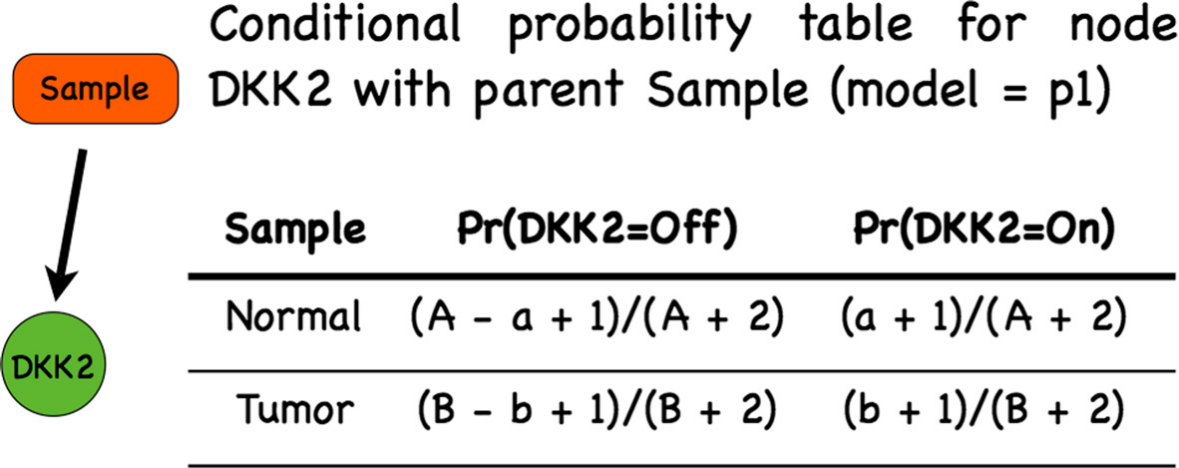
Conditional probability table for node *D* *K* *K*2 in $\mathcal {M}_{PBK+EI}$ and $\mathcal {M}_{PBK}$

Figure 8 shows the pictorial representation of one of the cpt in $\mathcal {M}_{\text {PBK+EI}}$ and $\mathcal {M}_{\text {PBK}}$.

#### DACT3 in $\mathcal {M}_{\text {PBK+EI}}$ (t1)

The conditional probability value for a gene being active or inactive is estimated from generated frequency estimates that can help derive probability values. These frequencies depict the following cases: (a) gene repressed in normal (represented by vector offINn), (b) gene expressed in normal (represented by vector onINn), (c) gene repressed in tumorous (represented by vector offINt), and (d) gene expressed in tumorous (represented by vector onINt) cases. For every *j*th entry in the vecTraining, if the label (labelTraining(j)) is normal (≤0) and the *D* *A* *C* *T*3 gene expression (vecTraining(j)) is less than the estimated median (≤vecmedian), then the value in vecTraining(j) is appended to offINn. Here, expression level lower than median indicates probable gene repression in the normal case. If the label (labelTraining(j)) is normal (≤0) and the *D* *A* *C* *T*3 gene expression (vecTraining(j)) is greater than the estimated median (≥vecmedian), then the value in vecTraining(j) is appended to onINn. Here, expression level greater than median indicates probable gene activation in the normal case. If the label (labelTraining(j)) is tumorous (≥0) and the *D* *A* *C* *T*3 gene expression (vecTraining(j)) is less than the estimated median (≤vecmedian), then the value in vecTraining(j) is appended to offINt. Here, expression level lower than median indicates probable gene repression in the tumor case. And finally, if the label (labelTraining(j)) is tumorous (≥0) and the *D* *A* *C* *T*3 gene expression (vecTraining(j)) is greater than the estimated median (≥vecmedian), then the value in vecTraining(j) is appended to onINt. Here, expression level greater than median indicates probable gene activation in the tumorous case. 



Lets again see how (1) the probability table would look like and (2) the probability table is stored in BNT [4], before estimating the values for cpt of *D* *A* *C* *T*3. Table 14 represents the conditions of *Sample*, *H*3*K*4*m* *e*3, and *H*3*K*4*m* *e*3 the probable beliefs of events (*D* *A* *C* *T*3 being on/off). Finally, from biological data presented in [2], the conditional probability values for the *D* *A* *C* *T*3 gene being active based on the histone modification and the available samples suggest that *D* *A* *C* *T*3 expression is high in normal samples when the histone repressive mark *H*3*K*27*m* *e*3 is reduced and the activating mark *H*3*K*4*m* *e*3 is present in high abundance. Thus, the probability, i.e., Pr(*D* *A* *C* *T*3=*a* *c* *t* *i* *v* *e*|*H* *K*327*m* *e*3=*l* *o* *w*,*H*3*K*4*m* *e*3=*h* *i* *g* *h*,*S* *a* *m* *p* *l* *e*=*n* *o* *r* *m* *a* *l*) is the fraction of the number of 1’s in the normal samples (a) and the total number of normal samples (A). For all other conditions of *H*3*K*27*m* *e*3 and *H*3*K*4*m* *e*3 when the *Sample* is normal, the probability of *D* *A* *C* *T*3 being active is (A-a), i.e., flip or complementary of Pr(*D* *A* *C* *T*3=*a* *c* *t* *i* *v* *e*|*H* *K*327*m* *e*3=*l* *o* *w*,*H*3*K*4*m* *e*3=*h* *i* *g* *h*,*S* *a* *m* *p* *l* *e*=*n* *o* *r* *m* *a* *l*). This is because in all other conditions of the histone marks, the probability of *D* *A* *C* *T*3 being active will be reverse of what it is when *H*3*K*27*m* *e*3 is reduced and *H*3*K*4*m* *e*3 is present in abundance. Similarly, in case of tumorous samples, the probability of *D* *A* *C* *T*3 being active will occur when *H*3*K*27*m* *e*3 is reduced and *H*3*K*4*m* *e*3 is high abundance (a rare phenomena). Thus, the probability, i.e., Pr(*D* *A* *C* *T*3=*a* *c* *t* *i* *v* *e*|*H* *K*327*m* *e*3=*l* *o* *w*,*H*3*K*4*m* *e*3=*h* *i* *g* *h*,*S* *a* *m* *p* *l* *e*=*t* *u* *m* *o* *r* *o* *u* *s*) is the fraction of the number of 1’s in the tumorous sample (b) and the total number of tumorous samples (B). For all other conditions of *H*3*K*27*m* *e*3 and *H*3*K*4*m* *e*3 when the *Sample* is tumorous, the probability of *D* *A* *C* *T*3 being active is (B-b), i.e., flip or complementary of Pr(*D* *A* *C* *T*3=*a* *c* *t* *i* *v* *e*|*H* *K*327*m* *e*3=*l* *o* *w*,*H*3*K*4*m* *e*3=*h* *i* *g* *h*,*S* *a* *m* *p* *l* *e*=*t* *u* *m* *o* *r* *o* *u* *s*). The reason for flip is the same as described above.

**Table 14:** Conditional probability table for *D* *A* *C* *T*3 in $\mathcal {M}_{\text {PBK}}$ (model - t1)

CPT for *D* *A* *C* *T*3 in $\mathcal {M}_{\text {PBK+EI}}$(model - t1)				
*H*3*K*27*m* *e*3	*H*3*K*4*m* *e*3	*Sample*	Pr(*D* *A* *C* *T*3=Off)	Pr(*D* *A* *C* *T*3=On)
1	1	Normal	h (1)	l (9)
2	1	Normal	h (2)	l (10)
1	2	Normal	l (3)	h (11)
2	2	Normal	h (4)	l (12)
1	1	Tumor	h (5)	l (13)
2	1	Tumor	h (6)	l (14)
1	2	Tumor	l (7)	h (15)
2	2	Tumor	h (8)	l (16)

h - probability of event being high; l - probability of event being low. 1 - low; 2 - high. Serial numbers in brackets represent the ordering of numbers in vectorial format

**Fig. 9:**
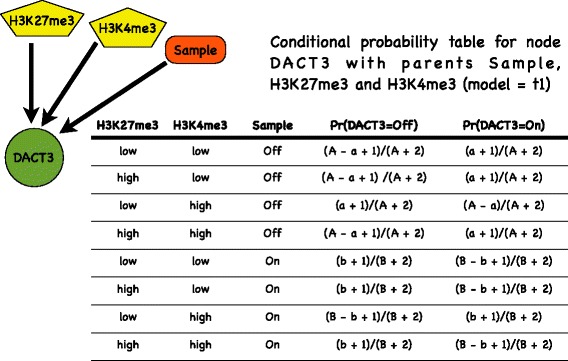
Conditional probability table for node *D* *A* *C* *T*3 in $\mathcal {M}_{\text {PBK+EI}}$

After the values in T have been established, a constant 1 is added as pseudo-count to convert the distribution to a probability distribution via Dirichlet process. Finally, the frequencies in T are normalized in order to obtain the final conditional probability values for *D* *A* *C* *T*3. Figure 9 shows the pictorial representation of one of the cpt in $\mathcal {M}_{\text {PBK+EI}}$.

Finally, for every gene, after the computation of the probability values in their respective cpt, the function generateGenecpd returns the following arguments as output.



Tables 8 and 9 from [1] show the assumed and computed estimates for all the nodes that represent non-genetic and genetic factors in the modeled pathway driven by the dataset. It might be that the probability values deviate from the mathematics formulations as these formulations do not capture all the intricacies of the biological phenomena. For example, the cross talk that happens between the histone modifiers varies the expression of *D* *A* *C* *T*3. But these time-varying dynamics cannot be captured in the model as the model represents a static time snapshot of the phenomena. More detailed explanation of this phenomena is available in [2]. The cpd for *D* *A* *C* *T*3 in Table 9 states that when *H*3*K*27*m* *e*3 is low and *H*3*K*4*m* *e*3 is high, irrespective of the state of the sample, the belief represented by the conditional probability that *D* *A* *C* *T*3 is repressed or off is high (and vice versa). Figure 9 shows the mathematical representation of the same. Similar interpretations can found for other cases.

## A programming project for practice

To get a feel of the project, interested readers might want to implement the following steps when the evidence eviDence is “me.” The code needs to be embedded as a case in the *switch* part of the twoHoldOutExp function. The idea is to perturb the methylation nodes with binary values and find if one can converge to the correct prediction of state of *TRCMPLX* as well as the *Sample*. These binary values are stored in a vector and represents a permutation of the methylation states of the methylation node in $\mathcal {M}_{\text {PBK+EI}}$. Varying the values of the vector can help study how perturbations affect the predictions from the network. The steps are given below:

1. Define variables for storing predictions of *TRCMPLX* (tempTRCMPLX) and *Sample* (tempSample).
2. Find the total number of methylation cases in $\mathcal {M}_{\text {PBK+EI}}$ and store the number in a variable noMethylation.
3. Generate binary values for noMethylation nodes. Define a cell (binaryStatesOfMethylation) that can store vectors of binary values where every permutation represents a set of methylation states. The total number of permutations should be 2^noMethylation^ (stored in noMethylationConfig). One might want to use quantizer and num2bin functions from Matlab.
4. Next, generate methylation evidences. Define a 2D matrix variable methylationEvidence that stores the methylation evidences. One might want to use the Matlab function str2num. Finally, add a value of 1 to methylationEvidence as the BNT takes in “1” and “2” as states representing binary values.
5. Build evidence for inference for every test example. The following steps might be necessary
  For every methylation configuration and for every methylation node, build evidence.
  Build a new Bayesian network in bnetEngine using jtree_inf_engine and store the modified engine (in engine) using the function enter_evidence.
  Finally, compute the Pr(*TRCMPLX* = 2 |ge as evidence) and Pr(*Sample* = 2 |ge as evidence) using the function marginal_nodes.
6. Store predicted results on observed methylation in structure Runs indexed with runCnt.

After the section of new code is filled in, run the code and check the results.

## Conclusions

A pedagogical walkthrough of a computational modeling and simulation project is presented using parts of programming code interleaved with theory. The purpose behind this endeavor is to acclimatize and ease the understanding of beginner students and researchers in transition, who intend to work on computational signaling biology projects. To this end, static Bayesian network models for the Wnt signaling pathway have been selected for elucidation. Due to the paucity of available manuscripts that explain the computational experiments from a tutorial perspective because of unwanted restrictive policies, this endeavor is a small step in this direction.
